# Metagenomic identification of novel viruses of maize and teosinte in North America

**DOI:** 10.1186/s12864-022-09001-w

**Published:** 2022-11-23

**Authors:** Ryan R. Lappe, Manjula G. Elmore, Zachary R. Lozier, Georg Jander, W. Allen Miller, Steven A. Whitham

**Affiliations:** 1 Department of Plant Pathology, Entomology and Microbiology, Iowa State University, 2213 Pammel Dr, Ames, IA 50011-1101 USA; 2 Boyce Thompson Institute, 533 Tower Rd, Ithaca, NY 14853 USA

**Keywords:** Mastrevirus, Tombusvirus, Umbravirus, Betaflexivirus, Maize chlorotic mottle virus

## Abstract

**Background:**

Maize-infecting viruses are known to inflict significant agronomic yield loss throughout the world annually. Identification of known or novel causal agents of disease prior to outbreak is imperative to preserve food security via future crop protection efforts. Toward this goal, a large-scale metagenomic approach utilizing high throughput sequencing (HTS) was employed to identify novel viruses with the potential to contribute to yield loss of graminaceous species, particularly maize, in North America.

**Results:**

Here we present four novel viruses discovered by HTS and individually validated by Sanger sequencing. Three of these viruses are RNA viruses belonging to either the *Betaflexiviridae* or *Tombusviridae* families. Additionally, a novel DNA virus belonging to the *Geminiviridae* family was discovered, the first *Mastrevirus* identified in North American maize.

**Conclusions:**

Metagenomic studies of crop and crop-related species such as this may be useful for the identification and surveillance of known and novel viral pathogens of crops. Monitoring related species may prove useful in identifying viruses capable of infecting crops due to overlapping insect vectors and viral host-range to protect food security.

**Supplementary Information:**

The online version contains supplementary material available at 10.1186/s12864-022-09001-w.

## Background

*Zea mays* L. (maize) is grown all over the world for uses as food, feed, industrial feedstocks, and ethanol production. Numerous plant viruses infect maize, some of which are attributed to significant yield losses by themselves or in concurrent infections with other viruses [1]. For example, maize streak virus (MSV) [2, 3], and more recently the maize lethal necrosis disease (MLND), caused by co-infections of maize chlorotic mottle virus and a potyvirus, such as sugarcane mosaic virus [4], have been devastating to maize production in Africa. Recently, high throughput sequencing (HTS) was used to identify viruses in maize leaves displaying symptoms consistent with maize lethal necrosis, and surprisingly, the spectrum of viruses occurring in co-infections causing this disease was expanded [5]. These results imply that breeding maize for resistance to MLND may not be as straightforward as expected. They also illustrate the power of using HTS to identify viruses infecting maize.

In more general terms, identification of novel plant viruses has improved dramatically with the advent of HTS technologies and the accompanying informatics pipelines for identification [6]. Application of HTS has culminated in the identification of an astounding number of unique viruses in recent years and a greater appreciation for the presence of plant viruses in natural and agricultural ecosystems [7, 8]. Plant viruses often cause symptoms, but they are also found more frequently than expected in asymptomatic plants [9]. This suggests that greater efforts are needed to better understand the viruses associated with crop and allied species that comprise different agroecosystems [10]. Furthermore, viruses causing asymptomatic infections or residing in weedy species near or mixed with crops may represent previously unappreciated threats to agriculture. Improving crop yield by mitigating pathogen-associated losses is expected to be increasingly important with climate change, population growth, and resource limitations [11]. Attempts to counter virus-associated crop loss have influenced breeding strategies to develop resistant lines [3, 12]. These efforts, while slow, have proven relatively successful [13]. However, the virosphere is constantly evolving, producing new potential threats [14]. Aside from their importance in causing disease losses, plant viruses, or sequences derived from them, can also be developed into powerful biotechnology tools for silencing [15], over-expressing [16], and editing plant genes [17–19]. Novel viruses may possess properties that provide new opportunities for such biotechnology applications or simply represent improvements over known viruses.

In addition to virus identification, HTS has also provided surveillance capabilities that enable researchers to track virus mutations and movement to assess potential threats related to virus evolution and epidemiology [20]. Such large-scale sequencing efforts are generally performed in regard to arboviruses [21] or potential zoonotically transferred viruses toward human protection efforts [22]. Identification of pathogens impacting crops and crop-related species is imperative to assess, control, and protect agronomically important crops, indirectly protecting humans by preserving the food supply [23].

Toward this objective, a metagenomic approach utilizing HTS was used to identify novel viruses of symptomatic and asymptomatic graminaceous species related to and including maize. We performed RNAseq on a total of 151 maize, teosinte, sorghum, switchgrass, and maize-associated aphid field-samples from Mexico and the USA. While teosinte and switchgrass are not staple crop species, they are related grass species, as such they may be reservoirs harboring viruses capable of infecting maize [24, 25]. In addition to known viruses that were identified, maize and teosinte samples evaluated by RNAseq were found to contain four novel viruses: three RNA viruses belonging to the *Betaflexiviridae* or *Tombusviridae* families, and a DNA virus belonging to the *Geminiviridae* family, which is to our knowledge the first *Mastrevirus* identified in North American maize.

## Materials and methods

### Plant material

**Fig. 1:**
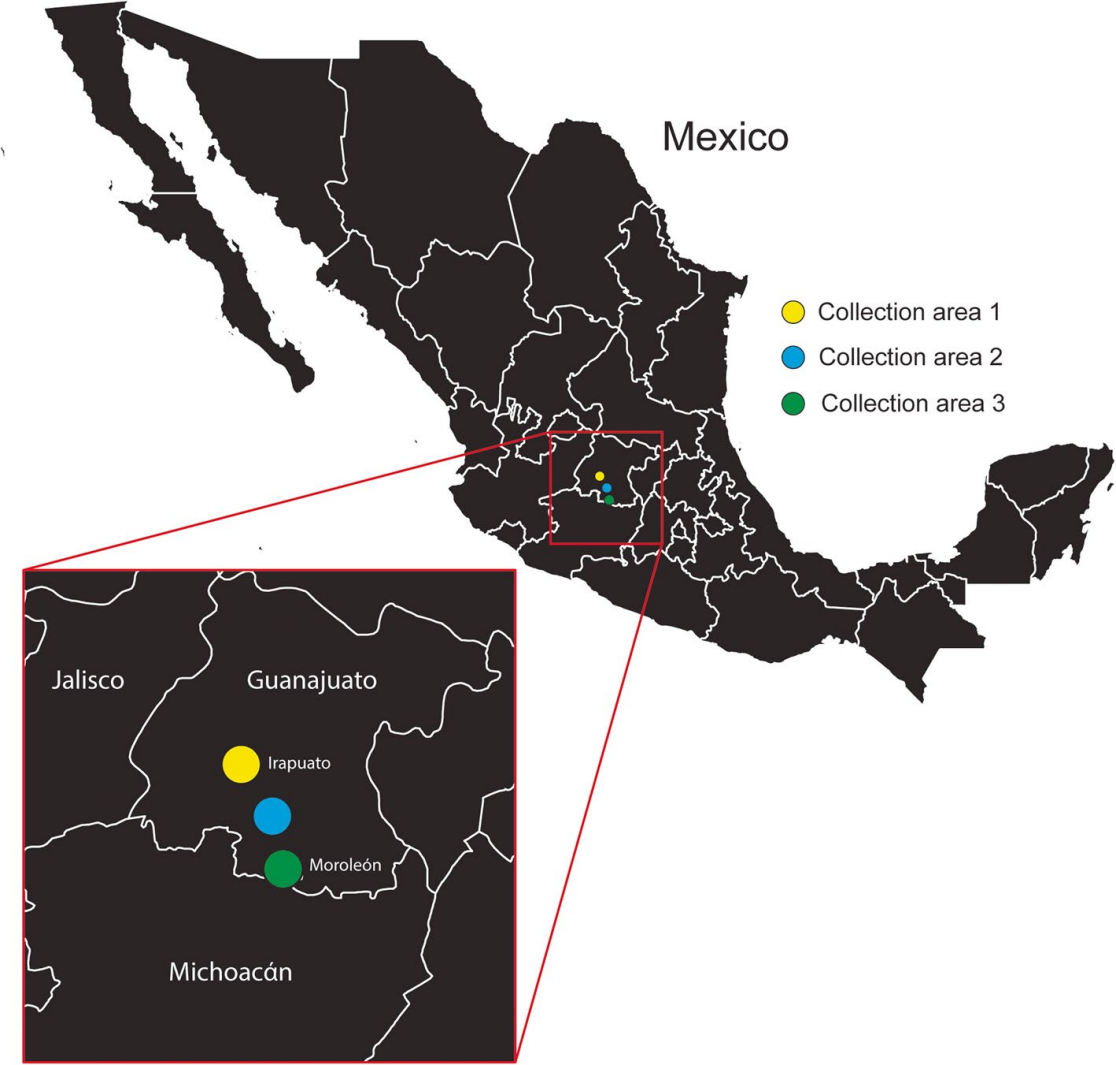
Leaf sample collection map of Mexico. Leaf samples originating from Mexico (Supplementary Table 1) were collected at varied GPS locations within collection areas shown. Collection areas 1 and 3 surrounded the cities of Irapuato and Moroleón, respectively

The majority of maize and teosinte leaf samples were collected in the state of Guanajuato, Mexico, in areas between and surrounding the cities of Irapuato and Moroleón (Fig. 1, Supplementary Table 1). Approximately 20 cm long leaf tips from mature maize plants were collected with scissors in October, 2017. Individual leaves were placed into 50 mL screw-cap centrifuge tubes. The tubes were transported as airline baggage at ambient temperature and then frozen at − 80 °C approximately three days after collection. Maize leaves were imported into the USA under USDA-APHIS permit PCIP-17-00464. Additional leaf samples from asymptomatic maize plants that were infested with aphids were collected within Iowa, Illinois, Indiana, North Carolina, and South Dakota in the USA (Supplementary Table 1). Leaf tissues were stored at − 80 °C prior to RNA isolation.

### RNA extraction, cDNA library preparation, and RNA sequencing

Leaf tissues were frozen in liquid nitrogen and pulverized using a Qiagen Tissuelyser II. Total RNA was extracted using the Zymo Direct-zol RNA Miniprep Plus kit (Zymo Research, Irvine, CA, USA) that included an on-column DNase treatment following the manufacturer’s specifications. The DNA-free total RNA was subjected to ribosomal RNA depletion by Illumina Ribo-Zero rRNA Removal Kit (Plant Leaf) (Illumina, San Diego, CA, USA) and concentrated using the Zymo RNA Clean & Concentrator-5 kit (Zymo Research, Irvine, CA, USA). Total RNA was quantified with a Qubit 3.0 fluorometer and quality assessed with a NanoDrop 2000. All RNA had A260/280 and A260/230 ratios between 1.55–2.09 and 0.55–2.29, respectively. Strand-specific cDNA libraries were prepared from ribosomal depleted RNA using the NEBNext Ultra II Directional RNA Library Prep kit (New England Biolabs, Ipswich, MA, USA). Illumina sequencing was performed on the dual-indexed cDNA libraries on an Illumina HiSeq3000 (paired-end 150 bp reads; 2 lanes per library).

### Next-generation sequencing (HTS) data analysis

Paired-end reads generated were evaluated for their quality by FastQC (Phred quality score above 30) [26]. Adapter removal and trimming were performed using Trimmomatic software [27]. All filtered reads were first mapped to plant and aphid genomes as reference using Hisat2 alignment program [28]. Reference genome sequences used for all maize samples included B73 (GenBank accession number: GCA_000005005.6), CML247 (GenBank accession number: GCA_002682915.2), and W22 (GenBank accession number: GCA_001644905.2). Reference genome sequences used for all teosinte samples included *Zea mays ssp. mexicana* (GenBank accession numbers: GCA_002813485.1, GCA_002813485.1, and GCA_000223545.1). Aphid and bacterial symbiont reference sequences used include the following: NC_002528.1 *Buchnera aphidicola* str. APS (*Acyrthosiphon pisum*) chromosome, complete genome, NZ_CP002701.1 *Buchnera aphidicola* str. G002 (*Myzus persicae*), complete genome, buchnera_scaf.v1.fa, *Acyrthosiphon pisum*, whole genome shotgun sequence, and *Myzus persicae* strain clone G006, whole genome shotgun sequence. The remaining unmapped reads were aligned to reference viral and viroid genomes (30,646 genome sequences) obtained from NCBI GenBank database (https://www.ncbi.nlm.nih.gov/genome/viruses) using Bowtie2 aligner [29]. The mapped viral reads and unmapped non-plant, non-viral reads were de novo assembled separately with a default k-mer of 25 using Trinity v2.6.6 [30]. All assembled contigs were queried against the all-organism NCBI nucleotide and protein database through BLASTN and BLASTX searches with default parameters using Blastplus v2.7.1 [31]. A similar informatics pipeline has previously been proven capable of virus identification by our research group [32].

### Novel virus validation

Following identification of putative novel viruses (contigs larger than 1500 bp with less than 80% sequence identity by BLASTN and BLASTX analysis) the original tissue corresponding to the RNAseq samples were utilized for DNA or RNA extraction dependent on the viral genome. DNA extraction followed the CTAB method as described [33], and RNA extraction was performed by TriZol (Thermo Fisher Scientific, Waltham, MA, USA) extraction method as recommended by the manufacturer. Isolated RNA was dsDNase treated and cDNA was generated with the Maxima H Minus cDNA synthesis kit (Thermo Fisher Scientific, Waltham, MA, USA).

The sequences of de novo assembled contigs were utilized to design primers suitable for PCR or RT-PCR amplification of DNA or RNA viruses, respectively (Supplementary Table 2). Amplification was performed using Platinum SuperFi II PCR Master Mix (Thermo Fisher Scientific, Waltham, MA, USA), a high-fidelity DNA polymerase, according to manufacturer’s protocol in presence of the included GC-enhancer. Each resulting amplicon covered approximately 25–100% of the putative viral sequence. Viral genomes amplified by multiple primer sets included overlapping areas of sequence to bolster confidence in sequencing. Amplicons were gel purified and extracted using the NEB Monarch DNA Gel Extraction Kit (New England Biolabs, Ipswich, MA, USA) and cloned into the pUC19 plasmid for Sanger sequencing with M13 primers [34] and internal viral genome primers (Supplementary Table 2). Sanger sequencing was performed by the Iowa State University DNA Facility. Sequences submitted to GenBank for all novel viruses reported are a combination of HTS data with sequence validation or correction by Sanger sequencing (Supplementary File S1).

### Phylogenetic analysis

The predicted amino acid sequences encoding the RNA-dependent RNA polymerase (RdRP) for each of the novel Tombusvirids and the Betaflexivirid were aligned to RdRP amino acid sequences from selected viruses in the *Tombusviridae* and *Betaflexiviridae* families using muscle 3.8.31 [35]. Multiple sequence alignments were passed to FastTree 2.1.11 with the “-lg” option [36]. Phylogenetic trees were drawn in FigTree 1.4.4 where they were re-rooted on the appropriate outgroups.

The complete nucleotide sequence of the novel *Mastrevirus* was aligned to selected *Mastrevirus* genomes using SDT 1.2 [37] which also produced the sequence identity heatmap. The values used to produce the heatmap shown in Fig. 4 are provided in (Supplementary Table 3).

## Results and discussion

**Fig. 2:**
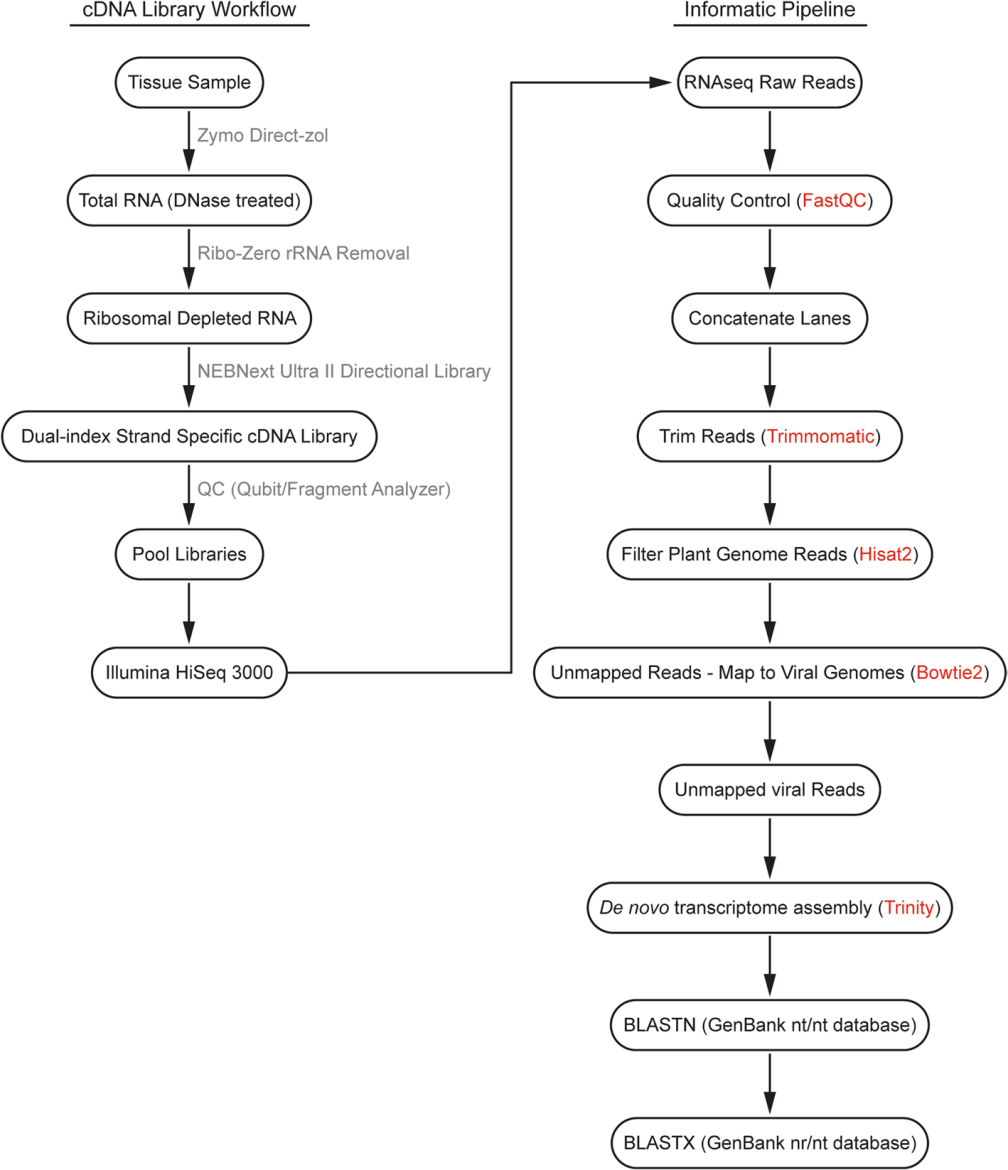
RNAseq library creation and informatics workflow. The cDNA library workflow (left panel) illustrates the process utilized to generate cDNA libraries for novel virus identification. Sequencing reads were analyzed using the informatics pipeline depicted (right panel). Intermediates shown in gray indicate names of kits used to perform the particular step. Red text identifies the informatics module used for the particular step in the pipeline

A total of 90 maize, 54 teosinte, 3 switchgrass, 1 sorghum, and 3 maize-associated aphid samples originating in North America were collected for RNAseq analysis with the goal of identifying novel viruses. Most of these samples were collected in areas between and surrounding the cities of Irapuato and Moroleón in the state of Guanajuato, Mexico (Fig. 1, Supplementary Table 1). However, 28 of the samples were collected in the USA. Leaf or aphid samples were processed through cDNA library creation for Illumina sequencing, and the resulting sequence data were analyzed using an informatics pipeline designed to identify known or novel viruses (Fig. 2).

These analyses are limited to near full-length or full-length viral genomes as opposed to fragments. Due to the large volume of samples and contigs identified as viral, for identification of novel viruses, we chose to focus on contigs greater than 1500 bases with less than 80% predicted homology to known plant-infecting viruses based on BLASTn and BLASTx searches to reduce the potential for false identifications. Numerous samples contained contigs that are viral in nature, but unlikely to be associated with plant-infecting viruses due to their sequence identity to viruses known to infect mammalian, insect, fungal, or bacterial hosts; thus they are not discussed here.

### Known viruses

Contigs associated with known viruses were identified in 17 of the 151 samples analyzed (Supplementary Table 1). Maize chlorotic mottle virus (MCMV) was found in 8 samples, maize yellow mosaic virus (MaYMV) in 4 samples, and maize rayado fino virus (MRFV) in 7 samples. Two of these samples contained a mixture of MaYMV and MRFV, and one had a mixture of MCMV and MRFV. All of the above samples were from Mexico. Of the 28 samples collected in the USA, one from South Dakota contained the complete tripartite genome of brome mosaic virus (BMV). None of the other USA samples had full-length viral genomes, despite the fact that most samples were infested with known aphid vectors (*Rhopalosiphum padi* or *R. maidis*). Most Mexican samples had disease symptoms while those from the USA did not. The presence of these viruses is no surprise. BMV is long known in South Dakota and MCMV and MRFV are endemic in Mexico [1]. While MaYMV per se may not have been reported in Mexico, it is also known as Maize yellow dwarf virus-RMV-II [38] and is extremely closely related to Maize yellow dwarf virus-RMV, which was known previously as Barley yellow dwarf virus-RMV [39]. The yellow dwarf viruses of cereals are well-known to occur in Mexico and worldwide [40].

### Novel viruses

#### Mastrevirus

Plant-infecting DNA viruses belonging to the family *Geminiviridae,* are small monopartite or bipartite circular ssDNA viruses. Members of this family belong to one of nine genera based on genome structure and organization, plant host, and insect vector [41]. The *Mastrevirus* genus are monopartite single-stranded DNA (ssDNA) viruses, most of which have monocot host plants [42]. The virion-sense of the *Mastrevirus* genome encodes movement (MP) and capsid (CP) proteins, while the anti-sense encodes the two replication associated proteins RepA (C1) and Rep (C1:C2), with Rep being the translation product of a splicing variant of *RepA* [43, 44]. *Mastrevirus* genomes also contain two intergenic regions, a long intergenic region (LIR) and a short intergenic region (SIR). The LIR contains transcription start sites and the origin of virion strand replication (*v-ori*) with a conserved nonanucleotide stem-loop motif 5′-TAATATTAC-3′ [45, 46], while the SIR contains transcription termination sites and the complementary-strand replication origin [47]. Historically these viruses have been identified within old-world continents such as Africa, Asia, and Europe, with more recent discoveries occurring in the Americas [48–50]. However, no maize-infecting *Mastrevirus* has been reported in North America.

A BLASTn search against the GenBank nr database using a 3137 base contig from maize sample #76 (Supplementary Table 1) identified a 168-nucleotide region with ~ 81% identity to the Rep coding sequence (CDS) of sugarcane striate virus (SCStV), a *Mastrevirus* [51]. Subsequent evaluation of this contig by a BLASTx search revealed potential homology (~ 31% query coverage with 39–44% amino acid identity) to the RepA proteins of SCStV and maize streak virus (MSV), which is the type member of the *Mastrevirus* genus. The putative proteins encoded by the open reading frames (ORFs) of the unknown virus also have homology to conserved *Geminiviridae* motifs in movement protein (MP), coat protein (CP), and replication protein (REP) (pfam01708, pfam00844, pfam08283, pfam00799), as well as a protein of unknown function, DUF1069, encoded in the MSV genome (pfam06370) (e-values 0–3.71e^− 5^).

To validate the presence and sequence of this putative *Mastrevirus*, DNA was isolated from maize sample #76 for amplification and sequencing of the viral genome (Supplementary Fig. 1, Supplementary Table 2). The resulting 2760-nt genome sequence confirmed the circular nature of the viral genome and corrected the original contig to obtain the full-length viral sequence that we designate, provisionally, as North American maize-associated *Mastrevirus* (NAMaMV) (GenBank accession: MZ852895).

**Fig. 3:**
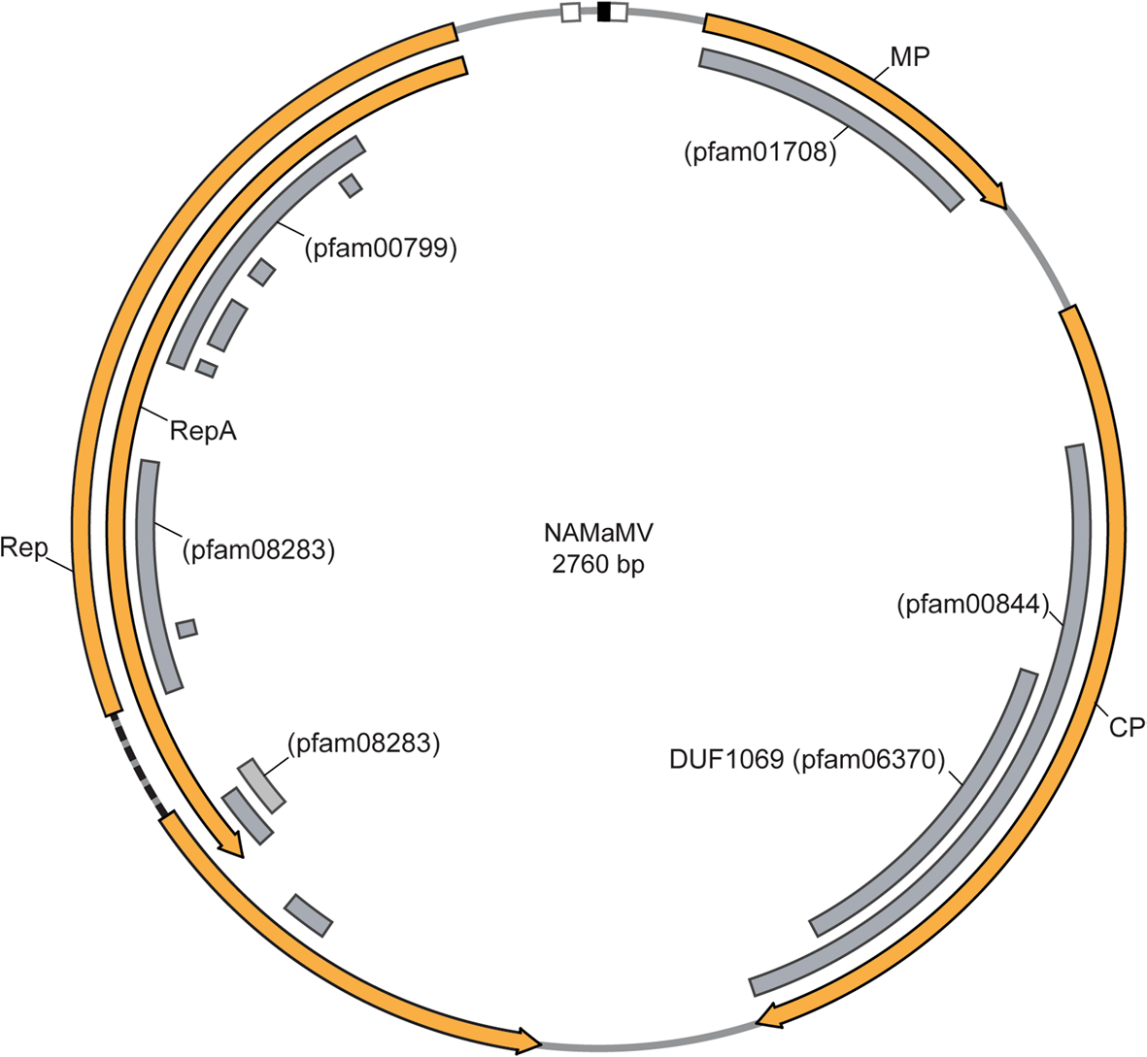
Organization of the putative novel NAMaMV genome. The NAMaMV genome was evaluated for conserved motifs by BLASTx (shown as gray boxes with corresponding pfam identification numbers). Open reading frames (orange arrows) and the Rep intron (black dashed line) were inferred from long open coding sequences and the presence of identified motifs. Additional information regarding identified motifs is provided in Supplementary Figs. 2 and 3

**Fig. 4:**
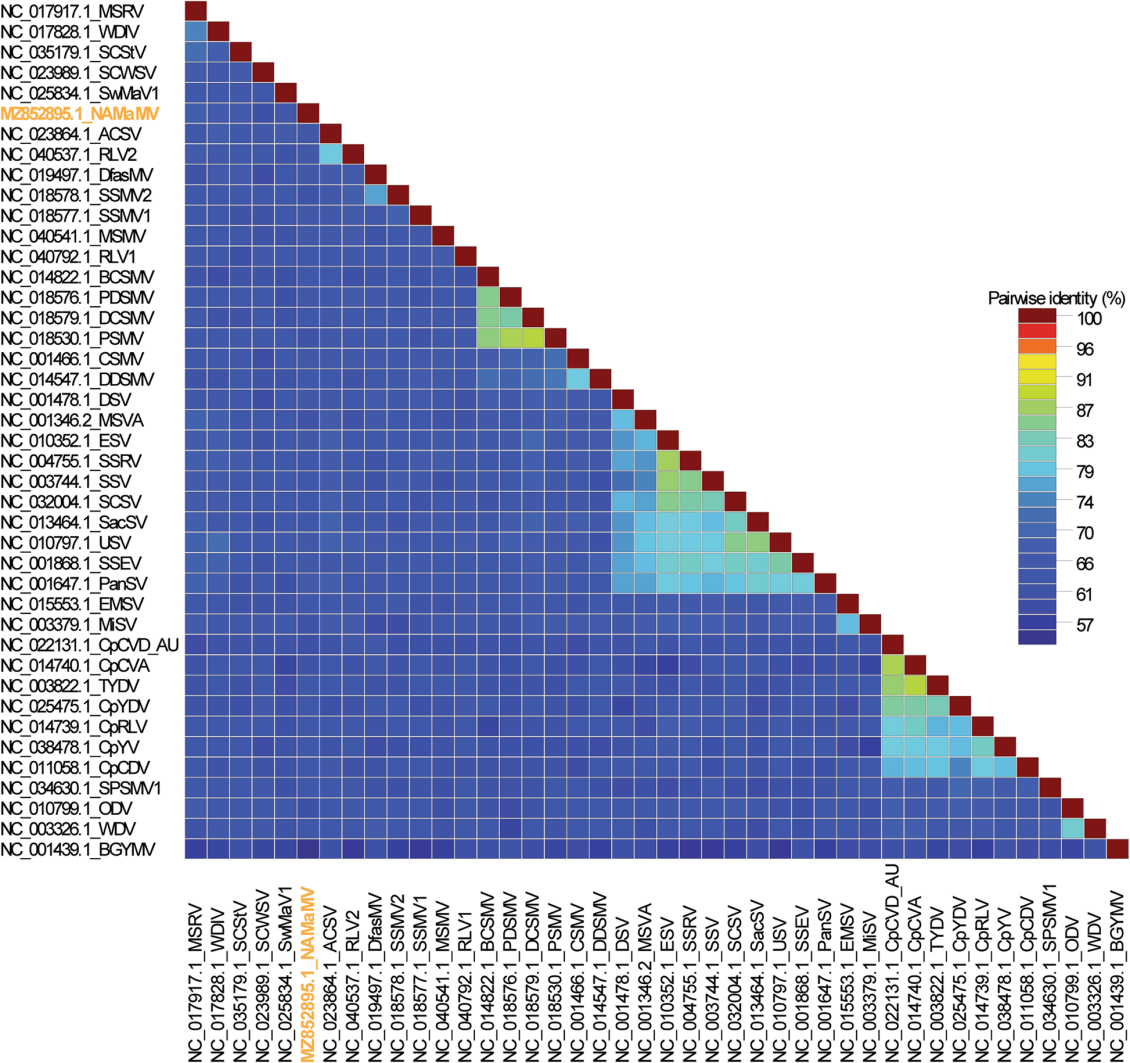
NAMaMV sequence demarcation tool analysis. The NAMaMV genome was compared to known *Geminivirus* genome sequences using the species demarcation tool (SDT) set forth by the International Committee on Taxonomy of Viruses (ICTV) for pairwise identity comparison to establish novelty. NAMaMV is highlighted in orange

The full NAMaMV genome contains putative ORF products with homology to known *Mastreviruses* identified by BLASTx (Fig. 3, Supplementary Fig. 2, Supplementary Fig. 3). Residing within the putative Rep proteins are conserved motifs such as rolling-circle replication (RCR) motif I – III [52], Geminivirus rep sequence (GRS) [53], LxCxE retinoblastoma binding [54], and dNTP binding domains [55] (Supplementary Fig. 3). The intron associated with the Rep C1:C2 transcript is presumed from the split Rep catalytic motif (pfam08283) (Supplementary Fig. 3). Full-length genome analysis to validate novelty was performed using the pairwise identity Sequence Demarcation Tool (SDT) set forth by the International Committee on Taxonomy of Viruses (ICTV) [37]. Novel species are required to possess less than 78% sequence identity to any recognized species. Our SDT analysis indicates NAMaMV is a novel member of the *Mastrevirus* genera of the *Geminiviridae*, with a maximum of 63.5% pairwise identity to *Maize streak Réunion virus* (MSRV) (Fig. 4, Supplementary Table 3).

The presumed LIR of NAMaMV includes the *v-ori* containing a non-canonical nonanucleotide motif 5′-TAATGTTAC-3′, differing from the canonical 5′-TAATATTAC-3′ sequence associated with members of *Geminiviridae* [41]. This nonanucleotide sequence variation has recently been identified in a watermelon-infecting isolate of a *Mastrevirus,* chickpea chlorotic dwarf virus (CpCDV) [56]. Thus, NAMaMV is the first monocot-infecting *Mastrevirus* with this nonanucleotide variation, as well as the first identified *Mastrevirus* in maize in North America.

#### Betaflexivirus

**Fig. 5:**
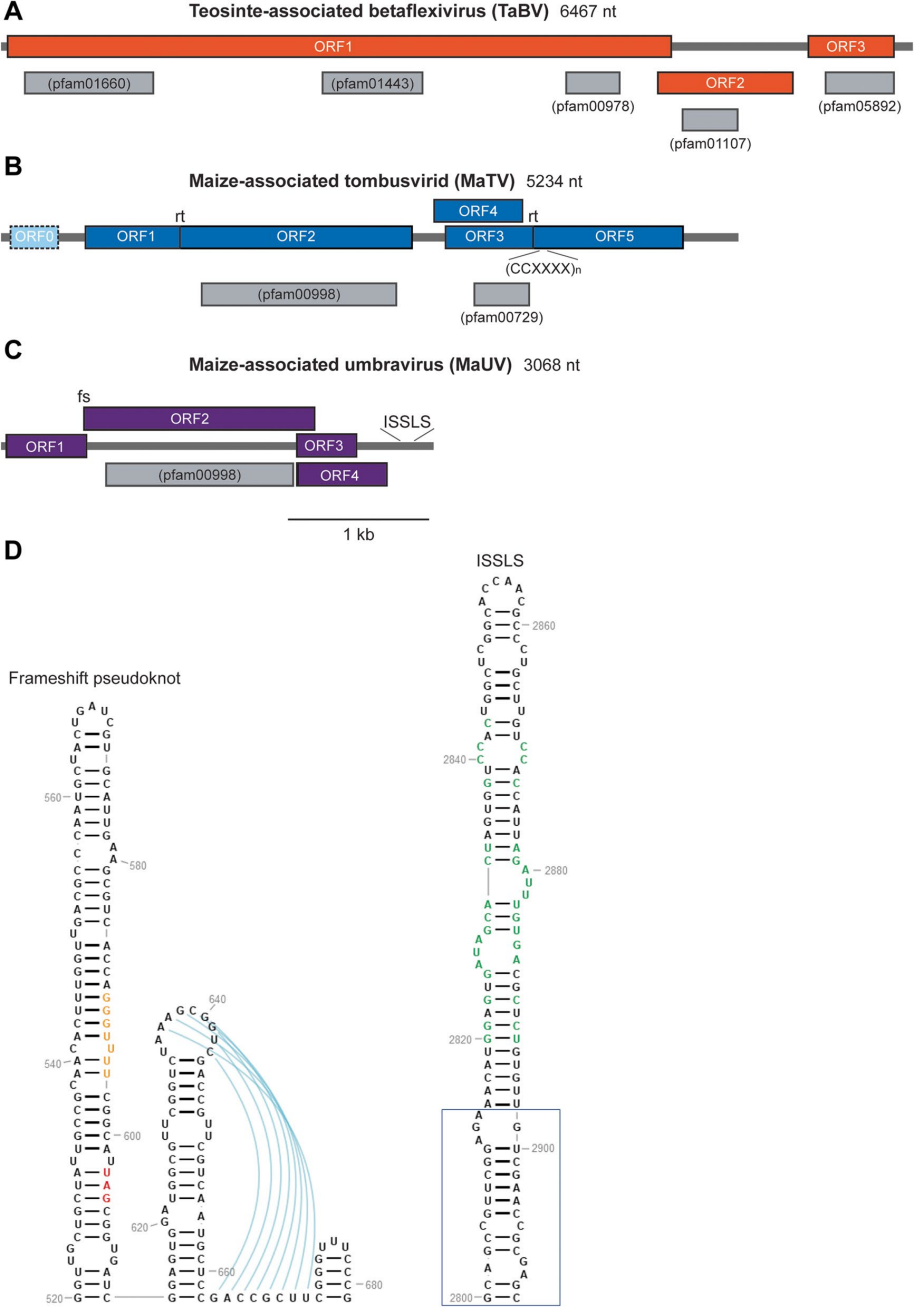
Organization of putative novel RNA virus genomes. **A** Putative TaBV genome map. Orange boxes indicate locations of predicted ORFs 1–3. **B** Putative MaTV genome map. Blue boxes indicate locations of predicted ORFs 1–5. The light blue box with a dashed outline depicts the location of ORF0, a possible but unlikely ORF. Locations of predicted in-frame readthroughs (rt) and the (CCXXXX)_n_ motif are labeled above and below the genome map, respectively. **C** Putative MaUV genome map. Purple boxes indicate locations of predicted ORFs 1–4. The location of a predicted frameshift element (fs) and ISSLS, which is predicted to enhance cap-independent translation [58], are indicated above the genome. **D** RNA secondary structure predictions for MaUV. The MaUV genome sequence was used to predict secondary structure of the putative − 1 frameshift pseudoknot and ISSLS using mfold [59] and visual inspection. The structures were drawn using RNA2Drawer [60]. Bases in orange and red represent the shifty site and the ORF1 stop codon, respectively.  Bases in green are conserved in other ISSLS [58]. Validated novel virus genomes were evaluated for conserved motifs by BLASTx. Identified motifs are depicted as gray boxes with corresponding pfam identification numbers inside or below. Open reading frames were inferred from long open coding sequences and the presence of identified motifs. Viral genomes are depicted relative to the scale bar equal to 1 kilobase (kb)

Members of the *Betaflexiviridae* family have flexuous filamentous virions with genomes of 6–9 kb coding for three to six genes depending on the genus. In general, these viruses contain a short 5′-UTR followed by ORF1, the largest gene product, which encodes the replication protein that possesses methyltransferase (Mtr), helicase (Hel), and RdRP functions [57]. Contigs associated with teosinte samples #49 and #106 were identified by a BLASTn search against the Genbank non-redundant database as having similarity (81.6% identity to a 125-base tract) to apple chlorotic leaf spot virus. Analysis of these contigs by BLASTx revealed putative amino acid similarity to a number of members of *Betaflexiviridae*, most significantly scaevola virus A (40.8% identity to an 832-base tract), and identified conserved motifs for MP, Mtr, CP, Hel, and RdRP (pfam01107, pfam01660, pfam05892, pfam01443, pfam00978) (e-values 6.8e^− 18^ – 6.2e^− 4^) (Fig. 5A). Comparison of the two novel contigs revealed they are approximately 85% identical to each other at the nucleotide level. The predicted translation product of the identified ORF1 sequence is 94% identical between these contigs. The ICTV species and genus demarcation criteria require novel species to have less than 72% nt (or 80% aa) identity for RdRPs, as such, these contigs appear to be different isolates of the same novel virus.

Tissue associated with sample #49 was utilized for RNA isolation, cDNA synthesis, RT-PCR, and sequencing of the amplicon to validate the presence and sequence of the virus (Supplementary Fig. 1, Supplementary Table 2) designated, provisionally, herein as teosinte-associated betaflexivirus (TaBV) (GenBank accession: OK018178).

**Fig. 6:**
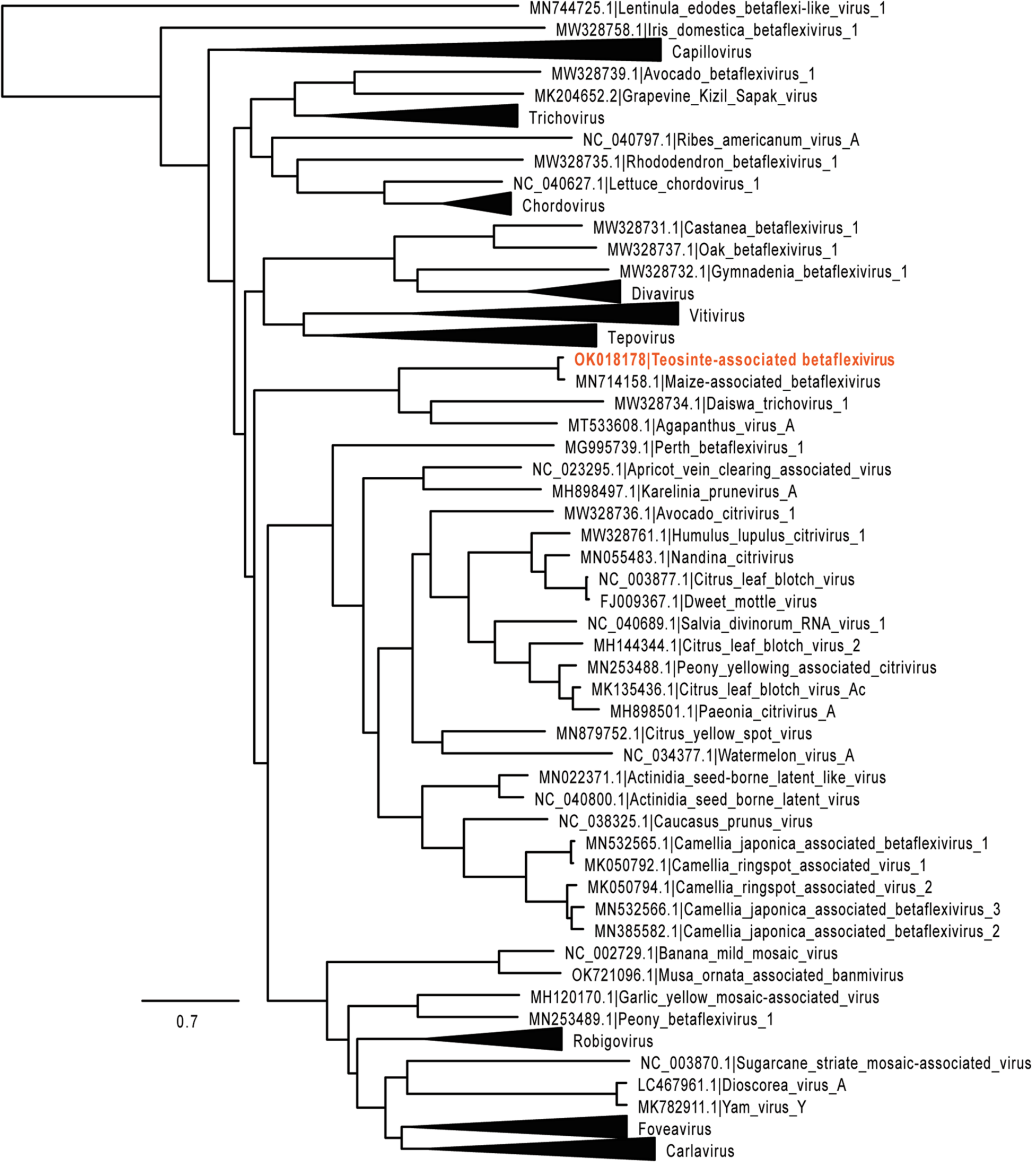
Phylogenetic analysis of the novel teosinte-associated betaflexivirus RdRP. The putative RdRP amino acid sequence for TaBV was aligned to RdRP amino acid sequences of selected viruses in the *Betaflexiviridae* family using muscle 3.8.31. Multiple sequence alignments were passed to FastTree 2.1.11. Phylogenetic trees were drawn in FigTree 1.4.4, where they were re-rooted on the appropriate outgroups. TaBV is color coded orange, as depicted in Fig. 5. The tree is drawn to scale with the scale bar representing substitutions per site. Names and representative GenBank accession numbers for viral sequences are listed

The presumed ORFs encoding the RdRP of TaBV was used for phylogenetic analysis using members of *Betaflexiviridae*. This phylogeny demonstrates TaBV RdRP similarity to members of *Betaflexiviridae* but it remains distinct from specific genera (Fig. 6), indicating TaBV is an unclassified member of *Betaflexiviridae*. The TaBV RdRP groups mostly closely with that of maize-associated betaflexivirus (MN714158.1), an incomplete viral genome sequence possibly of the same virus species as TaBV, but found in Rwanda [61].

#### Tombusviridae

*Tombusviridae* is a diverse viral family with genomes encoding four to seven ORFs [62]. Only ORFs 1 and 2 are translated from genomic RNA. The downstream ORFs are translated from subgenomic mRNAs often using a variety of noncanonical translation mechanisms [63]. The genomic and subgenomic mRNAs of tombusvirids are translated in a cap-independent manner, relying on a cap-independent translation element located in the 3’UTR [58, 64]. The distinguishing and unifying feature of *Tombusviridae* is the conserved RdRP translated from ORF2 by suppression of termination at a stop codon or by a − 1 frameshift to bypass a stop codon, generating fusions of the ORF1 and ORF2-encoded proteins [65]. Members of this family in the *Umbravirus* genus lack an ORF encoding CP and instead rely on a co-infection with a member of *Solemoviridae* to supply CP in trans for vector transmission [66].

Two contigs with 99% nucleotide identity were identified in maize and teosinte samples #3 (GenBank accession: OK018181) and #21 (GenBank accession: OK018182) (Supplementary Table 1), respectively. These contigs displayed sequence similarity to the 5′ UTR of Hubei tombus-like virus (~ 87% identity of 60 bases) by BLASTn search against the GenBank non-redundant database. A BLASTx search using these contigs showed 27% query coverage with 67% identity to the RdRP of apple tombus-like virus 2.

**Fig. 7:**
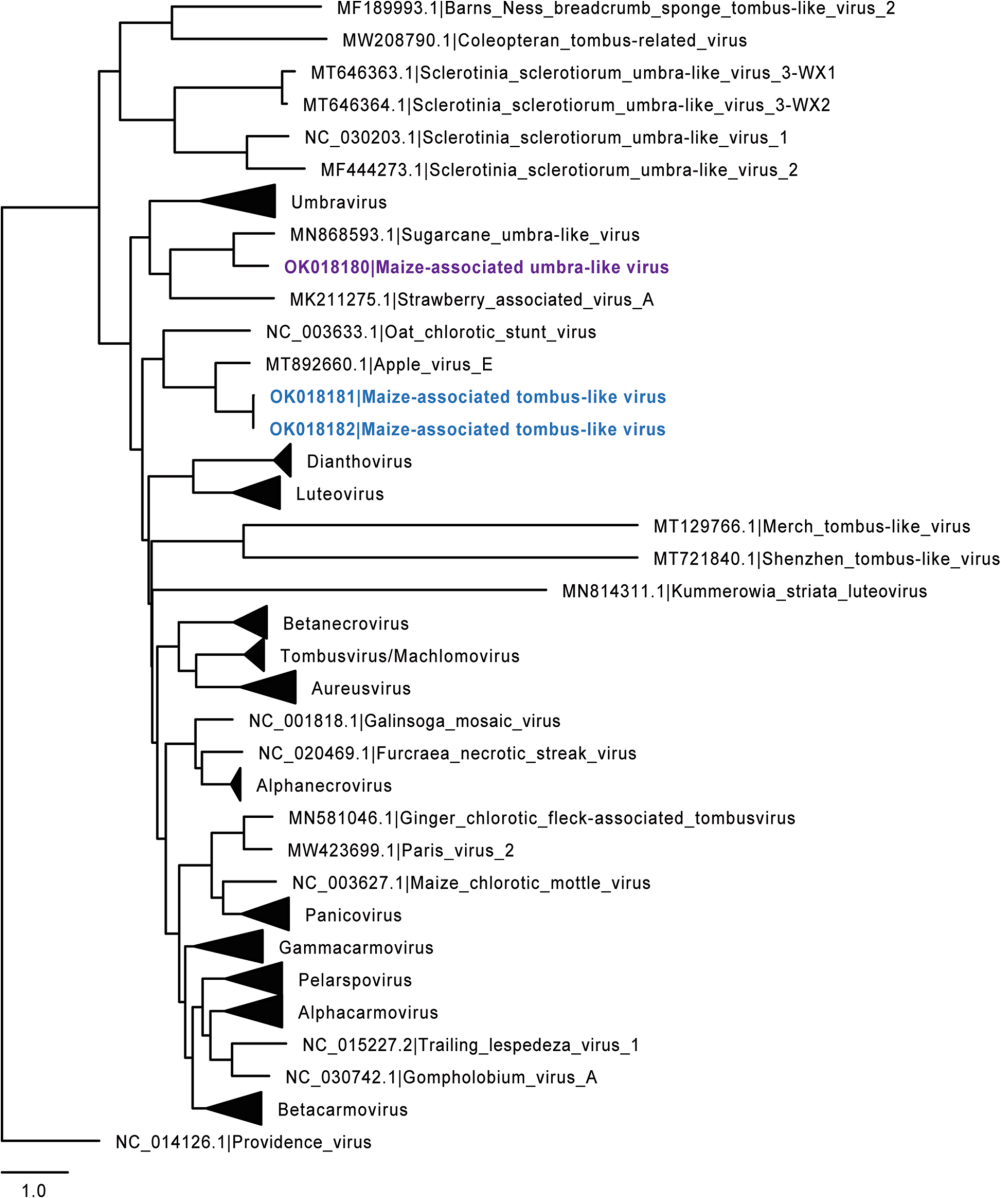
Phylogenetic analysis of the novel tombusvirus RdRPs. The putative RdRP amino acid sequences for MaTV and MaUV were aligned to RdRP amino acid sequences of selected viruses in the *Tombusviridae* family using muscle 3.8.31. Multiple sequence alignments were passed to FastTree 2.1.11. Phylogenetic trees were drawn in FigTree 1.4.4 where they were re-rooted on the appropriate outgroups. MaTV and MaUV are color coded blue and purple, respectively, as depicted in Fig. 5. The tree is drawn to scale with the scale bar representing substitutions per site. Names and representative GenBank accession numbers for viral sequences are listed

Subsequently, RT-PCR amplicons were sequenced to validate the presence of this virus, which is designated, provisionally, herein as Maize-associated tombusvirid (MaTV) (GenBank accessions: OK018181, OK018182) (Supplementary Fig. 1, Supplementary Table 2). The genome of this virus is predicted to encode at least five proteins, including conserved motifs within CP and RdRP (pfam00729 and pfam00998) (Fig. 5B). Phylogenetic analysis of the presumed RdRP with *Tombusviridae* members demonstrates that these viruses cluster within *Tombusviridae*. While they appear to belong to this family, they do not display significant homology to the members within any specific genus. Their closest relative is apple virus E (AVE, GenBank accession: MT892660), which also has not been assigned to a genus (Fig. 7).

Both MaTV and AVE appear to differ remarkably from other tombusvirids. Firstly, the replication protein encoded by ORF1 initiates 600 nt from the 5′ end of the genome, which is much longer than the 10 to 160 nt found in other tombusvirids. Secondly, in MaTV, there is a 112 codon ORF, upstream of ORF1, which we call ORF0. The potential protein product showed no homology to any known proteins, and this ORF is absent in AVE, which has no significant ORFs upstream of ORF1. Finally, if ORF0 is translated, it would be difficult to explain how ORFs 1 and 2 are initiated, given that ribosomes would terminate translation of ORF0 203 nt upstream of the ORF1/2 AUG. In summary, we speculate that the entire 600 nt upstream of the ORF1/2 AUG may be untranslated, which certainly appears to be the case with AVE. Such a long 5′ UTR might contain an internal ribosome entry site (IRES), as in picornaviruses or triticum mosaic virus (*Potyviridae*). Such long IRESes are highly structured, but we found few strong predicted secondary structures in this 600 nt tract, using structure prediction programs mfold and Scanfold [59, 67].

The genomic region downstream of ORF2 bears some resemblance to those of genus *Luteovirus* (*Tombusviridae*) and the *Polerovirus* and *Enamovirus* genera in the *Solemovirdae*. ORF3 encodes the coat protein (CP), immediately followed by ORF5 which is likely translated via in-frame readthrough of the ORF 3 stop codon. Shortly downstream of the ORF3 stop codon is a (CCXXXX)_4_-(ACXXXX)_1_-(CCXXXX)_6_ repeat that differs by one base from the (CCXXXX)_8–16_ repeat at this position that participates in readthrough of the ORF3 stop codon in the luteo/polero/enamoviruses [68]. Interestingly, AVE, in which ORF5 is also in-frame with ORF3, has no (CCXXXX)_n_ motifs whatsoever (Accession no. MT892660). Downstream of the (CCXXXX)_n_ repeats, the amino acid sequence of the MaTV readthrough domain (encoded by ORF5) shows little similarity to those of the luteo/polero/enamoviruses. Translation of ORF5 to generate the readthrough protein (RTP) extension to the C-terminus of the CP is required for the virion of luteo/polero/enamoviruses to be transmitted by aphids, in a circulative (but non-replicative) manner [69–71]. Thus, we predict that MaTV is also transmitted in this fashion by a sucking insect.

The luteo- and poleroviruses (but not enamoviruses) also encode a movement protein ORF (called ORF4) that overlaps entirely with the CP ORF. Here, we identified such an ORF in MaTV, but it is predicted to start upstream of ORF3, unlike in the luteo- and poleroviruses, in which ORF4 initiates a few codons downstream of the start of ORF3. The product of MaTV ORF4 (called P4) has no obvious sequence similarity to the P4 proteins of luteo- and poleroviruses, and yielded no Genbank hits using BLASTp. The AVE sequence has indels that place the first 30 codons, including the initiator methionine, out-of-frame with the rest of the ORF. Thus, the biological relevance of ORF4 is uncertain.

The luteoviruses and poleroviruses also encode a 45 amino acid, non-AUG-initiated ORF (ORF3a) required for movement that overlaps with the 5′ end of ORF3. We found some non-AUG-initiated ORFs in this vicinity, one of which is three times longer than ORF3a, but no ORFs showed homology to ORF3a. In summary, MaTV, and the previously sequenced AVE, appear to represent an unusual genus in the already diverse *Tombusviridae* family, characterized by an extremely long 5′ UTR, and an arrangement of ORFs around the CP ORF (ORF3) that imperfectly resemble that found in the *Luteovirus* genus.

Finally, BLASTn analysis of a 3068 bp contig associated with maize sample #5 (Supplementary Table 1) yielded a singular result with nominal similarity (~ 82% identity of a 67-base tract) to the CDS of the RdRP of carrot mottle virus (CMoV), an *Umbravirus*. Analysis by BLASTx indicated ORF similarity to the RdRP of umbra- and umbra-like viruses Ethiopia maize-associated virus and opuntia umbra-like virus, and identified the conserved RdRP motif (pfam00998, E-value: 1.7e^− 78^) in the putative ORF (Fig. 5C).

The 5′ UTR through ORF2 of the genome was amplified by RT-PCR and sequenced for validation of this sequence (Supplementary Fig. 1, Supplementary Table 2), we designate as maize-associated umbra-like virus (MaUV) (GenBank accession: OK018180). Phylogenetic analysis of the RdRP demonstrates homology sufficient to cluster this virus within *Tombusviridae*, however, not within the *Umbravirus* genus (Fig. 7), indicating that MaUV is an unclassified umbra-like virus, like those recently investigated [58, 72]. MaUV RNA shows strong structural similarities to those of the other umbra-like viruses, including a predicted large pseudo knotted structure proposed to induce − 1 frameshifting for translation of the RdRP [72], and a large, predicted bulged stem-loop resembling an “I-shaped structure-like structure” (ISSLS) (Fig. 5D), found in other umbra-like viruses [58], that would confer cap-independent translation of the viral genome.

## Conclusions

We have presented genome sequences of three novel RNA viruses, one belonging to *Betaflexivridae*, and two members of *Tombusviridae*, as well as a novel DNA *Mastrevirus* belonging to *Geminiviridae*. Each of these viruses was found within leaf tissue of maize or teosinte originating in North America and collected in the summer of 2017. All tissues collected yielded reads or contigs associated with viruses, many of which are known to infect maize, such as MCMV, MRFV, and MaYMV. Although leaf samples were collected throughout North America, the novel viruses were identified only from tissue collected in Mexico. We speculate this may be due to the significantly fewer number of samples originating from the USA, variations in susceptibility to pathogens in maize lines, diversity and abundance of virus-transmitting insects, differences in use of pesticides, as well as climate and geographic location differences that influence virus ecology. In addition, the USA samples did not display any virus-like symptoms although they were from plants that were infested with aphids.

Two isolates of the novel *Betaflexiviridae* member TaBV were discovered in teosinte samples geographically separated by 74 km. These contigs are approximately 85% similar at the nucleotide level, with 94% identity at the amino acid level for ORF1 encoding the viral replication protein. This would indicate that TaBV may have been widespread among teosinte in the area sampled.

The novel *Tombusvirid* MaTV was discovered in both teosinte and maize samples collected in the same geographic location. This emphasizes the necessity of identification of novel viruses of related species surrounding crops, as they are potential reservoirs of viruses with similar or overlapping host ranges. Owing to the dependence of umbraviruses on a helper virus in the *Solemoviridae* family to provide CP for encapsidation and transmission, we expected to identify a known or novel solemovirid associated with MaUV. However, there were no contigs associated with one in that sample, as such, we are unable to identify the viral partner that may be required for transmission of this virus.

The novel *Mastrevirus*, NAMaMV, is to our knowledge the first *Geminiviridae* member identified in maize in North America. Identification of a novel *Mastrevirus* in a crop species in North America is interesting as these are generally viewed as old world viruses, although a number have been identified in Central and South America in recent years. These recent discoveries raise questions as to their origins, whether these viruses were endemic and only being discovered due to technological advances, or are evolutionary products of old world mastreviruses.

We demonstrate here that metagenomic studies of crop and crop-related species are useful for the identification and surveillance of known and novel viral pathogens of crops. Monitoring related species may prove useful in identifying viruses capable of infecting crops due to overlapping insect vectors and viral host-range. This could provide a preemptive warning, affording researchers time to characterize these viruses and to diminish associated impacts through plant breeding or insect vector control programs to protect our food and energy security.

## Supplementary Information

**Additional file 1:** **Supplementary Table 1.** List of maize, teosinte, switchgrass, sorghum, and maize-associated aphid samples used in this study. Details of sample location, associated viruses identified with their GenBank accession numbers in field-grown maize, teosinte, switchgrass, sorghum, and maize-associated aphid samples across North America. The viruses were identified from the all-organism NCBI nucleotide and protein databases through BLASTN and BLASTX searches using Blastplus V2.7.1. GPS coordinates of collection correspond to the illustration in Fig. 1.

**Additional file 2:** **Supplementary Table 2.** Novel virus contig amplification and sequencing primers. Individual primer sequences used to produce and sequence viral amplicons in Supplementary Fig. 1.

**Additional file 3. Supplementary File 1. **Sequences of the novel viruses in FASTA format.

**Additional file 4:** **Supplementary Table 3.** SDT values derived from comparison of NAMaMV to Mastrevirus reference sequences. Raw data output corresponding to the heat map depicted in Fig. 4 for pairwise sequence comparison of the novel Mastrevirus NAMaMV to reference Mastrevirus genomes using SDT 1.2. The ICTV requires a novel Mastrevirus to possess a pairwise sequence identity value of less than 0.78 by comparison to any recognized species.

**Additional file 5:** **Supplementary Fig. 1.** Amplification of novel viruses identified by RNAseq for validation of presence and sequence. Nucleic acids were isolated from leaf samples identified by RNAseq to contain novel viruses. Resulting DNA or cDNA was used for amplification of viral sequences by primers designed from assembled contigs (Supplementary Table S2). Subsequent to amplification, the fragments of interest were gel extracted and cloned into pUC19 for Sanger sequencing.

**Additional file 6:** **Supplementary Fig. 2.** Annotation of putative nucleotide sequence elements of NAMaMV. The genome sequence of NAMaMV highlighted with corresponding labels and colors for open reading frame start and stop codons, as well as the presumed Rep-associated intron in orange text.

**Additional file 7:** **Supplementary Fig. 3.** Annotation of amino acid motifs predicted in putative NAMaMV open reading frames. Putative open reading frame translations are highlighted with corresponding labels and colors with pfam identification numbers identified by Blastx analysis.

## Data Availability

The datasets generated during the current study are available in the Sequence Read Archive (SRA accession: PRJNA753546). Sequences for the identified novel viruses NAMaMV, TaBV, MaTV, and MaUV can be found at the NCBI GenBank database under accession numbers MZ852895, OK018178, OK018181, and OK018180, respectively.

## Abbreviations

HTS: High throughput sequencing
RNAseq: RNA sequencing
MLND: Maize lethal necrosis disease
MCMV: Maize chlorotic mottle virus
MaYMV: Maize yellow mosaic virus
MRFV: Maize rayado fino virus
MP: movement protein
CP: Capsid protein
Rep: Replication protein
RepA: Replication associated protein
CDS: Coding sequence
SCStV: Sugarcane striate virus
MSV: Maize streak virus
NAMaMV: North American maize-associated Mastrevirus
LIR: Long intergenic region
Mtr: methyltransferase
Hel: Helicase
RdRP: RNA dependent RNA polymerase
TaBV: Teosinte-associated betaflexivirus
sgRNA: subgenomic RNA
MaUV: Maize-associated umbra-like virus
