# Supplementary material for: Metagenomic identification of novel viruses of maize and teosinte in North America
Source: BMC Genomics. 2022 Nov 23;23:767. doi: 10.1186/s12864-022-09001-w (PMC9685911; doi:10.1186/s12864-022-09001-w)
Supplement: Supplementary file 3 — Additional file 3. Supplementary File 1. Sequences of the novel viruses in FASTA format. [file 12864_2022_9001_MOESM3_ESM.rtf]

Supplementary File 1. Sequences of the novel viruses in FASTA format.

>OK018181.1 Maize-associated tombusvirus isolate P1A3S3, complete genome
GGCCCGGTGAGGTATTTTCACTCCATCTAGACTTCATGAAACCTTGATTGGTGGATTGTCGTGGGATGGG
GCAGAGTTCGCCACACGTAGCTTCACAGCGCGGACTGGTAATATGTGGAACTATCCTAGTTAAGGGGGGT
GCATACCTTAATTTGCTCACCCTGGTCCTTGGTAAGGTTGATGGACCCGGGAAGGAACCCGATACATTCC
GGACTTGCGAGAGTCCCGCGGCCGGCGTAATGGCGATTGAGGAAGGTAAAGCGCACCGTAAATGCGCAGT
GGGAGTGCACCTGATTACAAACTCACTGGATACGTTATGCGCGCGTACCAGTGTCCCACATCGCGTTACT
CAGCTTGGGAAACTGGGGCCTGTCCGTTTCGGATGGGTGTCAACCCCATAGGAAGGTAAGGCGCACCTTA
AATGCGCAATGGGAGTAAACCTAATTACAAATCTCTAGGTCAGAAGATCGAGCCACTACGTGCCGTGGCG
ATAAACTTGCATTGTGATGGAGATTTGGTCGCTAATACCTACCATCTAAATCCTCAAGTTGATGACCCGC
CTGGCCCCACGCTCGGGGCTAACCACAACCCTATCACTCAATCATGGCTACAACTACACAGAACCCCGAA
TTTAACTTGAATGCCCGAATTCAGCTTCTGCGTGTCTGCCCTGATGTTGTCTTCACTTCGTCTGAGCCTG
AAGTTGAGACGCCAGTTTTGGAGGAAGTTGCACCTTTGCCAGTGGTGCCGTCTCCCTCCATGCCGGCCCG
CCCGTCTTTCCCTTGCGGATTGCCGAAAGTGGACGTTGTGTCTGCCTTCCTTTCGTTCTTCCCGGCATAC
GTGCCAGCGGCAGTGCAACAGGAGGTAGATGTTCTAGTCCATCAGTACGACGCACCATGTGTGGACGAGG
CCAGGGACGGTTTGGATGTGGTAGGGACCAGCAGCGGGGCTGCTGGCGAGAAGTCAAAGCCGGTGCGCAA
GCCGCGTGCCGACAACTCCAGGTGGTGGGTGAATTACTCTCTGTTGGCTCATGCCGAGTTCAACAGCCCG
AAGTACACCCGTGCACAGGAGATGATTGTGTCCGCTTGGATTCGTAAGACGATGTCGTCTGACGGTGTTA
CAGGGCTACAGATCGCACAGGTCTTGGCCGCTGCCACCAGGATGGCATTTGTCCCGAATGCTTCGGACAT
CCTTGCTGCCCAGATTGGTTCAGTCCAATCCATCAAGGCTCGTGAGCGCCTCGTTGCCACCAGATGGTGG
AGTAGCTGGTGGGACAGCTATTCTGGCCGCTACGACAGTAGCGAATAGGGTGGCCCAGTGAGCGTACCAG
GGAGGGACGCGGCAGAGTCTGCCGCCCCACACCCTCCTAGTTTGGTGGTGCGCAATAAAGTGGGACACGC
CAACCGCGTTCGACGCACTTTCTTGGTGGGGCAAAGGATGTCCCCGCCGCGTCGTATCTACGCGTTCAAT
TCATCCTTGCAAAACCTAGTCAAAGGGGTGAAGGAGCGAGTCTTCTTCGTCAAAAGCCCCTCTGGTGGAT
TTGAGACCGTCCCGGCACCCTTGGACGGTATCGCTGAAGCCAGGCTTGGGCATCTCCGAGATACCTTGGT
GAGCTTGTGTCCACGTCTGTGCCCGTTGGAACGAGAGGAATACCCTCTCCAGTACACGGGTAAGAAACGG
GCCACATACACCAAGGCTGTGGAAGCTCTACGTAGTAGGGAGCTGACACGTAAGGACGCAGAGGTCACAA
TTTTCACAAAGACCGAGCGCACGCTCAAGCATGACGCGGTACCCCGGATCGTTTCGCCAATGAGTCCGGA
ATGCAATCTAGAGACGGGTAGGTTTGTGAAGCCTATGGAAGCCCCGATCTGTAAAGCCATTGCTGCTATG
GCTGGCCACACCGTTGTGATGAAGGGCAAAAATGCATCACAAATTGGTGACGTTCTTAAGCAGCATTGGG
ATGAGATGGGAGGTGACGGAGTATGCGTCGCTATCGGATTGGACGCGTCCAGGTTTGACCAGCATGTTTC
GAAGCAATTTTTGCAGTTCGAGCATACTCATTATCCGCCTTTGCTTATCAGTCCTTTGGACCGGGCTGAG
TGCAAGCGGTTGTTGAGTTGGCAGTGTGACACCACTGCCTTTGGTCGCACCTCGGATGGCACTGTCAAGT
ATTCAATTTCCGGTACACGCCTTTCTGGCGTGATCAATACCGGCCTTGGCAATTGTATCATAGCGTCCGA
GATGTGTATTGCGTATTGTCGCGAGCGTGGTGTAGATTTCCGCTTGGCCAACAATGGAGATGATTGTGTC
ATCTTCTTGAATAAGCGGGATCTTGCCGTGTTCAGCGACGGCCTCTCATTGTGGTTCAGGGAGATGGGAT
TCAACATGGTAGTGGAGGAGCCCGTATACGAGCTGGAGGAGGTTGTGTTTTGTCAGGCACAACCTGTCTT
CGACGGGCAATCCTGGGTCATGGTTCGTGACCCCAGGTCCGCTATTGCGAAGGATTGTGTTTCCTTGAAG
CCGTGGCGTAACGAGAAAGAGTATCAAAGCTGGATCAAGTCAGTTGGGATGTCGGGGCGCGCCTTAGCTG
GTGGCATCCCCATCTACAATGAGTTCTATTCCAGTTTTGTTCAAGCTGGTGGTGCAGCACGTCCCTTGTC
CATGGAGGACCCATCCGTTGGTGGTGGTCTCTATTGGATGTCTAAGGGTATGCACCGCCAGGGTTTGGCC
GTATCGGACCAGGCCCGCCTTTCGTTTTGGAAGGCGTTTGGCATCGACGCACAGATGCAACTCGAACTCG
AAAAACATTACGCCAACACCATTCCGAAATTTATTCCTGTAGAGAAAGTAGAAGATCTTGGGGACGTATA
CCCCACGATGGACTGCGACTATCTCAGGATGCTTCCCCCTTGCCAGGGTTAACCGGCAGCAATTGGGTTG
TAGGGACTAAGTGGGCCAAAACTGTGAAGCAGGTAACTGTGGACGCAAAACTTCAGTGCTAAATCGAGAA
ATCGTAAATGCCGAGAGACTGCACGGCTCAACCCCGCCGACGGGGTGCCCTATGATGTACAGTCCTGTTG
TGGTGGCAGGATCCAATACACACCACATTTACAAACTGTCGATCTCAATCACAATCAGCAATTCCACCGT
CATCATCGAGAATGGCGCGCAAGAATCAACAACAGAATGCCGCAAAGGCAATCAACCAAAAGAAGACCAA
AGCCAAAACCAAGTCTGCGGTCGTCCGTGCTCCAACTTCCATCTCTGTCAGAAATACCACCCAGAATTCT
GGGTCAAGGCAGGAGGTAATGATCGGAACGGTCACGAACGAAGCAAAAGTGCTCACCCTGCACTGCGCCA
ACATGCCGTGGTTGCAGGGGGTGGCTCCGTCATACCAGACTTGGAACATGAAAGACGTACGCGTCAAGTT
CGTACCCCGGATGTCGACAGCGACAAACGGGACGGTCTCGATGTGTTTTCTGAGGGACTTCGAGGACGCT
ACCCCGACGACTGTCGACCAAATGTCCCTAGTCCAGGGGACGGTCAGCGCGGCAGTCTGGGACAAGCTGA
ACCTCCGCGTCCCCGATCGAAAAGCAATGGCGTACTGCTCCCGCTCAAACTTTCTATTAATGGGCTCAAC
CGACAAAAGCGAGCGAGCCCTGGGGCGGATAGTAGTTGTGCCTGATACCGATCTTGACATCAAGGCCAAG
ATTGGGTACCTGTATGTGGAGTACACTCCGCAATTTACAGGACCAATCGACCCATTGCTACAAAAGGAAG
GCTAGGTCTCTGATAGCCCCACTACGACCGTACCAACTGAACCCACACAACCTGAACCTGGTCCACTACC
CGGGACTGATCCGACTCCAGAGCCTGAGCCGGATCCAGTACCGGAAGGTGAACAGTACTCCACACCCACC
AAGAAAGCGATGTTCAACGCCTCCTGGGCGAATAACGATTTCCTTGGGTTGGGGTACTCTGCCAGGATCA
CCACGAATTTTGAATATATGCACAGCGGCGCCGGACTCACAGAGAACGGTTGGGTGTTGCAAAACAACCA
TGCTGAGGCTGCTATCAACATTTTCGTGGAATTGATTATTCACATCAAAGGTTATAAGGGTTTCTATTGG
AAGAAGGGTGGTTACCTTCAACTTACGGATAGTGTCGCTATCGACTATCAACCAAGCTGGCGCGACGGCG
CGGGGAGGGTGGTATGCGTCTGGAGTGGCACCCTGTGGCCCAACTCTTGGATAGCATTGTGGTACAACAA
CGACGCTGAGTCCGGTACAACAGAGCAACCTGTTGGCGCTTACGTCGAATGTTACGTCCGGGAGATCATT
GGCAACGAAGATGTCTCCCAGGCCATACCCACCAAACCCCCGTACCGTAGGAGAATCGGTCGTGATGAGC
CAAACGATGCCGATATGTACACATTTCCAGTCTCACTCCCACCTTCGGGTCTGGTGGACTTGGTGGCGTA
CAAGGGTTCCAACATCGTAAGGCTATATCATCAACTCTTGAATGAGTTCAATCCAGGAAGTGGTTCTGGG
TATAGGACGGGCTGGAGGTTTAGCGAGCCGGAGCACGATACGGTCACCAGGTCCATGTACCAGGAGAGCG
TCAATGGCAACGGTTATCTGTACCTTCCGGTCGGAAATCCTTCCTGGGATAGCAAACCGCATCCTATCAG
CTTCGAGATCGTGGGATCCAAGCTGGCATTGAAGTCTGAGAGGGGGCAATATCTCGCTTGGCCCACCACT
GGGTCTGTGGGAAATTACGATGGCGCGCTTGCTAGTTATGAGTTGGTTTACACTAATGCGATTTCTGAGT
CAATTTTGTGGGATGTTTCTGTGATTTCTTAATGGTTCGTAGTTTGCTTATTTCTTTAGTAGAATGCGTT
GGGCCGTATAGGCGCCGCACAAAGAGCAGGGTAGTGCATGAGGGGCGAGAGGACCCGTAATAGAAATCTC
GCCCGGCTGGAAGCCTGCGTTCCGCGTGCCCAACAAACGCCACATCTCCTCAGTGGCTAAACGAGGGAAA
AACAATGTTTCGTGGCGACGGTACACTTTACACCGTGGCTGGGCGCCAAACACCTGTAATTGTGCAGGGG
CCCAAATTTCGTGCTAACTTTCGTTGCGGGAGAGGGTGGAACCTCTTCTTCTTTTCTACTACGTGTCCAG
AGGGGCACCGAGGCTGGGGTCCCGTCCGCGAGGATGGGGGGCACCCACGAGTCT

>OK018180.1 Maize-associated umbra-like virus isolate P1A5S5, complete genome
GGAATTCGTACAGAATCAAGTGTCTACCCACCTTAGGATGTCCCGTGACCACGCACGAGAGATCCTCAAG
AACACTGCTCGTTACCCGTCGAATGTAGTACGATCAGCTTACAAATGGGTCGTTCGACGCCGTCCCCGCA
GTGTCTACACCTCCGCTGAGGCTGTTGGCACTGTTGTGGCGGGCTGCGTGAACAAGGTGCTGGACTTGAT
TCCCAGCGTCCATGATCGCGCAGCGGCTCGCGCCGCCGAGGCTGAAATTGACTGCCTCGTTAACGAGTGG
GTTCGTGGCTCTGTCAACTCGGACGAACACGCCGAAGTTGGTTGGGCACTCAAGCTCAGGGACCGTTACG
GTTTCCCTGCCGCTAGTGAGCCTACTAGGCTTACTGGTGAGAGATGGGTGCTCGAGCAACTACAGAAGGA
GGCTTCGGAGGAGCAGGCAATGAACTGGCGCGCTGAGACTGGGGAGACCTGGTCCCGCGTCCTCATTGCT
AAATCCGATCCCCTCGGTTGGCAGGGTAAGGTTGCTGCTATTGCCGCAACACTTTGGTTGACGCCCAATG
CTACTGATCGTGCATTGAAGCGTCACCAGGGTTTTCGGCATTAGCGGTGATCGGAGTGGATGGCGTTCGG
TCTAAAGCGGTCGACCGTTCGTCAATGCTCCGACCGCTTCGGGTTTCCCGGCCCCGACAAAGTCGACGGA
GATCACTGCAGCTACTATTACCCCTACCCCGCGCATGCTTTGTCAATCACGCCAATGATGGCGTTAACAT
GGTGCGGGTGCTCGAGAACCGGGTATTAAAGTACAAAGGCAGAGATCCTGTAAAGCCTCTGACTGAAGCC
ATAAGCAGATTGGACTCGGCGATTGCACGAAAATACAAGATCCAACCGAAACGCGTGCAACCACTGACGT
ATCAAGGCTTCCTCAATTGTTACCACGGACGCCGGCGCACGAGGTACGAACAAGCCGTGGAGCAATTGAG
TCGGCGCCCTCTCCAGCCGAAGGATTCACGGGTCGAAACTTTCATCAAGAATGAAAAATTCGACTGGTTG
TCCAAGAGAGCCGAGACCGACCCTCGAGCAATTCAGCCCCGAAAGCCCAAATTTTTGGCTGAAGTCGGTC
GATGGTTCAAGCCGCTTGAGCACATAATGTACCAAGACTTGGCAAAGCGGTTGTACGACAGCGACGAACC
TGTGATAGCTAAGGGCTTGAATGCAGAGCAGACTGGCGAGTTGCTCTGGCGCAAATGGAGTCGTTTCAAG
CGGCCTGTTTGCGTGTCCTTGGACGCTTCACGGTTCGATCTTCATGTCAGCCAGGACATGTTGAAGTGGA
CGCATCGCATGTACAATCGATATTGCCGGTCTAAAACGCTTAACAAGCTGCTATCTTGGACACTGACAAA
CCGTGGAACCGCCTCTTGCGCAGATTCTGCTTACCAGTATGAGGTTGAAGGCAGACGGATGAGTGGCGAT
ATGGACACCGCCCTCGGCAATTGCGTCATCATGACCTGTATCACGTGGTTCATTCTTAATGAACTCAACA
TTAAGCATGAACTGCTTGACAACGGGGATGATTGTCTCTTCATCTGCGAACTTGCCGATGCTCCAAGCGA
TGAGACCATTTCGAAGATGTATCTCGACTTTGGCTTCGAAGTGCGTTTGGAGTCTAGGAGCGAAGTGTTC
GAGCGCATAGAGTTTTGCCAAACCCAGCCAGTATGGCGTGGCGATTCATGGGTGATGATTAGGAATATCA
AGAGTTTAAGCAAGGACGTTACAGATGTCAATTGCGTGACAGACAATGCCTTCACCCATTGGCTGAAGGC
GGTTGGACTGTGCGGACGCGTGTTGAACTCAGGTATTCCCATCTTCCAATCGTTCCACAACATGCTTACT
AGGATTGGTACAGACTCGAGAATTAAGAACGCCGTGTACTATGAATGCGGCTTGGTTAACCTGATAAAAG
GGATGACAAGAGAGAAGCGCGAAATTGCGGATGAAACACGCCTTTCGTTTTACCGAGCGTTTGGAATTCC
TCCCCACCGTCAGATTGCCATTGAATCTTGGTATGACTCATTACAGGGCCCGATGGGTAAACTAAAGATT
CATGAATGGCAATTACAACTCAAAGAGGAATACGCATACGGAAGCGCGTGGTTCGAACGAGACGACGAAC
TGCTCCCCGCATTATGCGGCCCCCCCGCAATATGTGGAGGGAGTCGGGACTCGTCCTAGCCCCACTCTCT
GTCAAGAAGGTCAAGACGATGATGACCATGGAAACGTTTCTCCCCAACGACTTTCAACTGTCGAAGGAGG
CGATATACGTTACATGGCCTGTCAACGTCACCAGCTTTCCCACCCTGCTATCAGGGGTCACCAACGCTAC
TCACTGGCGCGTTACACGAGCAACCGTGGGTATGGAGCCCGCGTTGTCAACCAGTACCCAAATAGTAGGC
CTCGCCAACGCAGACGGAGCCTATTCTTATGGAACTGGTTACGGCGAAGTTTTCAAACGGTTGCGCGTGT
GTAATTACTCCTGGCGCTCACCGGTCGGAGGAAATGTTTCGGTGTCGTGGCCTATTAACATGGATTTCAT
CGAAAACGACGATGCCCACAAGAGTACTCTGCGAACAGTGCAATTCTTGCTGGCCGTCACCAACCCGGGT
GTGATATCAACGCAATCACAGAAACACACGGCTTGGGCGGAAATGCAACTCGAGCTGGAATGCATAGTTA
AGGCTACGTGATCTTCTGGGAGCTTGGCATCGACTGCCCCGTGGTGAGGCACACCCTCCTAGGTGAGTAG
CAGCCGTTCGGAGAAACATGGAGTGATAGCACTAGTGGTCCACTGGCTCGGCACCAACGCCCTGCTTGTC
CACCATTAGATTTGTGACGCTCTGTGTTGTCGAACCGCGAGCAACCACCTCAGGATGACCGTCTGGGCAA
CCAGACAACCCCTCGGGTAGATAATAGAGGGGGGTAACCGATCGCTTAGACTGCTGGATAGGATCGCCGG
TGTGTCACCGGGCCGATGACCCAGCAGGCTGCCCCGGGTAAACTAAAGATTCATGAAT

>OK018182.1 Maize-associated tombusvirus isolate P1B2S21, complete genome
GCCCGGTGAGGTATTTTCACTCCATCTAGACTTCATGAAACCTTGATTGGTGGATTGTCGTGGGATGGGG
CAGAGTTCGCCACACGTAGCTTCACAGCGCGGACTGGTAATATGTGGAACTATCCTAGTTAAGGGGGGTG
CATACCATAATTTGCTCACCCTGGTCCTTGGTAAGGTTGATGGACCCGGGAAGGAACCCGATACATTCCG
GACTTGCGAGAGTCCCGCGGCCGGCGTAATGGCGATTGAGGAAGGTAAAGCGCACCGTAAATGCGCAGTG
GGAGTGCACCTGATTACAAAATCGCTGGATACGTTATGCGCGCGTACCAGTGTCCCACATCGCGTTACTC
AGCTTGGGAAACTGGGGCCTGTCCGTTTCGGATGGGTGTCAACCCCATAGGAAGGTAAGGCGCACCTTAA
ATGCACAATGGGAGTAAACCTAATTACAAATCTCTAGGTCAGAAGATCGAGCCACTACGTGCCGTGGCGA
TAAACTTGCATTGTGATGGAGATTTGGTCGCTAATACCTACCATCTAAATCCTCAAGTTGATGACCCGCC
TGGCCCCACGCCTGGGGCTAACCACAACCCTATCACTCAATCATGGCTACAACTACACAGAACCCCGAAT
TTAACTTGAACGCCCGAATTCAGCTTCTGCGTGTTTGCCCTGATGTTGTCTTTACTTCGCCTGAGCCTGA
AGTTGAGACGCCAGTTTTGGAGGAAGTTGCACCTTTGCCAGTGGTGCCGTCTCCCTCCATGCCGGCCCGC
CCGTCTTTCCCTTGCGGATTGCCGAAAGTGGACGTTGTGTCTGCCTTCCTTTCGTTCTTCCCGGCATACG
TGCCAGCGGCAGTGCAACAGGAGGTAGATGTCCTAGTCCATCAGTACGACGCACCATGTGTGGACGAGGC
CAGGGACGGTTTGGATGTGGTAGGGACCAGCAGCGTGGCTGCTGGCGAGAAGTCAAAGCCGGTGCGCAAG
CCGCGTGCCGACAACTCCAGGTGGTGGGTGAATTACTCTCTGTTGGCTCATGCCGAGTTCAACAGCCCGA
AGTACACCCGTGCACAGGAGATGATTGTGTCCGCTTGGATTCGTAAGACGATGTCGTCTGACGGTGTCAC
AGGGCTACAGATCGCACAGGTCTTGGCCGCTGCCACCAGGATGGCATTTGTCCCAAATGCTTCGGACATC
CTTGCTGCCCAGATTGGTTCAGTCCAATCCATCAAGGCTCGTGAGCGCCTCGTTGCCACCAGATGGTGGA
GTAGCTGGTGGGACAGCTATTCTGGCCGCTACGACAGTAGCGAATAGGGTGGCCCAGTGAGCGTACCAGG
GAGGGACGCGGCAGAGTCTGTCGCCCCACACCCTCCTAGTTTGGTGGTGCGCAATAAAGTGGGACACGCC
AACCGCGTTCGACGCACTTTCTTGGTGGGGCAAAGGATGTCCCCGCCGCGTCGTATCTACGCGTTCAATT
CATCCTTGCAAAACCTAGTCAAAGGGGTGAAGGAGCGAGTCTTCTTCGTCAAAAGCCCCTCTGGCGGATT
TGAAACCGTCCCGGCACCCTTGGACGGTATCGCTGAAGCCAGGCTTGGGCATCTCCGAGATACCTTGGTG
AGCTTGTGTCCACGTCTGTGCCCGTTGGAACGAGAGGAGTACCCTCTCCAGTACACGGGTAAGAAACGGG
CCACATACACCAAGGCTGTGGAAGCTCTACGTAGTAGGGAGCTGACACGTAAGGACGCAGAGGTCACAAT
TTTCACAAAGACCGAGCGCACGCTCAAGCATGACGCGGTACCCCGGATCGTTTCGCCAATGAGTCCGGAA
TGCAATCTAGAGACGGGTAGGTTTGTGAAGCCTATGGAAGCCCCGATCTGTAAAGCCATTGCTGCTATGG
CTGGCCACACCGTTGTGATGAAGGGCAAAAATGCATCACAAATTGGTGACGTTCTTAAGCAGCATTGGGA
TGAGATGGGAGGTGACGGAGTATGCGTCGCTATCGGATTGGACGCGTCCAGGTTTGACCAGCATGTTTCG
AAGCAATTTTTGCAGTTCGAGCATACTCATTATCCGCCTTTGCTTATCAGTCCTTTGGACCGGGCTGAGT
GCAAGCGGTTGTTGAGTTGGCAGTGTGACACCACTGCCTTTGGTCGCACCTCGGATGGCACTGTCAAGTA
TTCAATTTCCGGTACACGCCTTTCTGGCGTGATCAATACCGGCCTTGGCAATTGTATCATAGCGTCCGAG
ATGTGTATTGCGTATTGTCGCGAACGTGGTGTAGATTTCCGCTTGGCCAACAATGGAGATGATTGTGTCA
TCTTCTTGAATAAGCGGGATCTTGCCGTGTTCAGCGACGGCCTCTCATTGTGGTTCAGGGAGATGGGATT
CAACATGGTAGTGGAGGAGCCCGTATACGAGCTGGAGGAGGTTGTGTTTTGTCAGGCACAACCTGTCTTC
GACGGGCAATCCTGGGTCATGGTTCGTGACCCCAGGTCCGCTATCGCGAAGGATTGTGTTTCCTTGAAGC
CGTGGCGTAACGAGAAAGAGTATCAAAGCTGGATCAAGTCAGTTGGGATGTCGGGGCGCGCCTTAGCTGG
TGGCATCCCCATCTACAATGAGTTCTATTCCAGTTTTGTTCAAGCTGGTGGTGCAGCACGTCCCTTGTCC
ATGGAGGACCCATCCGTTGGTGGTGGTCTCTATTGGATGTCTAAGGGTATGCACCGCCAGGGTTTGGCCG
TATCGGACCAAGCCCGCCTTTCGTTTTGGAAGGCGTTTGGCATCGACGCACAGATGCAACTCGAACTCGA
AAAACATTACGCCAACACCATTCCGAAATTTATTCCTGTAGAGAAAGTAGAAGATCTTGGGGACGTATAC
CCCACGATGGACTGCGACTATCTCAGGATGCTTCCCCCTTGCCAGGGTTAACCGGCAGCAATTGGGTTGT
AGGGACTAAGTGGGCCAAAACTGTGAAGCAGGTAACTGTGGACGCAAAACTTCAGTGCTAAATCGAGAAA
TCGTAAATGCCGAGAGACTGCACGGCTCAACCCCGCCGACGGGGTGCCCTATGATGTACAGTCCTGTTGT
GGTGGCAGGATCCAATACACACCACATTTACAAACTGTCGATCTCAATCACAATCAGCAATTCCACCGTC
ATCATCGAGAATGGCGCGCAAGAATCAACAACAGAATGCCGCAAAGGCAATCAACCAAAAGAAGACCAAA
GCCAAAACCAAGTCTGCGGTCGTCCGTGCTCCAACTTCCATCTCTGTCAGAAATACCACCCAGAATTCTG
GGTCAAGGCAGGAGGTAATGATCGGAACGGTCACGAACGAAGCAAAAGTGCTCACCCTGCACTGCGCCAA
CATGCCGTGGTTGCAGGGGGTGGCTCCGTCATACCAGACTTGGAACATGAAAGACGTACGCGTCAAGTTC
GTACCCCGGATGTCGACAGCGACAAACGGGACGGTCTCGATGTGTTTTCTGAGGGACTTCGAGGACGCTA
CCCCGACGACTGTCGACCAAATGTCCCTAGTCCAGGGGACGGTCAGCGCGGCAGTCTGGGACAAGCTGAA
CCTCCGCGTCCCCGATCGAAAAGCAATGGCGTACTGCTCCCGCTCAAACTTTCTATTAATGGGCTCAACC
GACAAAAGCGAGCGAGCCCTGGGGCGGATAGTAGTTGTGCCTGATACCGATCTTGACATCAAGGCCAAGA
TTGGGTACCTGTATGTGGAGTACACTCCGCAATTTACAGGACCAATCGACCCATTGCTACAAAAGGAAGG
CTAGGTCTCTGATAGCCCCACTACGACCGTACCAACTGAACCCACACAACCTGAACCTGGTCCACTACCC
GGGACTGATCCGACTCCAGAGCCTGAGCCGGATCCAGTACCGGAAGGTGAACAGTACTCCACACCCACCA
AGAAAGCGATGTTCAACGCCTCCTGGGCGAATAACGATTTCCTTGGGTTGGGGTACTCTGCCAGGATCAC
CACGAATTTTGAATATATGCACAGCGGCGCCGGACTCACAGAGAACGGTTGGGTGTTGCAAAACAACCAT
GCTGAGGCTGCTATCAACATTTTCGTGGAATTGATTATTCACATCAAAGGTTATAAGGGTTTCTATTGGA
AGAAGGGTGGTTACCTTCAACTTACGGATAGTGTCGCTATCGACTATCAACCAAGCTGGCGCGACGGCGC
GGGGAGGGTGGTATGCGTCTGGAGTGGCACCCTGTGGCCCAACTCTTGGATAGCATTGTGGTACAACAAC
GACGCTGAGTCCGGTACAACAGAGCAACCTGTTGGCGCTTACGTCGAATGTTACGTCCGGGAGATCATTG
GCAACGAAGATGTCTCCCAGGCCATACCCACCAAACCCCCGTACCGTAGGAGAATCGGTCGTGATGAGCC
AAACGATGCCGATATGTACACATTTCCAGTCTCACTCCCACCTTCGGGTCTGGTGGACTTGGTGGCGTAC
AAGGGTTCCAACATCGTAAGGCTATATCATCAACTCTTGAATGAGTTCAATCCAGGAAGTGGTTCTGGGT
ATAGGACGGGCTGGAGGTTTAGCGAGCCGGAGCACGATACGGTCACCAGGTCCATGTACCAGGAGAGCGT
CAATGGCAACGGTTATCTGTACCTTCCGGTCGGAAATCCTTCCTGGGATAGCAAACCGCATCCTATCAGC
TTCGAGATCGTGGGATCCAAGCTGGCATTGAAGTCTGAGAGGGGGCAATATCTCGCTTGGCCCACCACTG
GGTCTGTGGGAAATTACGATGGCGCGCTTGCTAGTTATGAGTTGGTTTACACTAATGCGATTTCTGAGTC
AATTTTGTGGGATGTTTCTGTGATTTCTTAATGGTTCGTAGTTTGCTTATTTCTTTAGTAGAATGCGTTG
GGCCGTATAGGCGCCGCACAAAGAGCAGGGTAGTGCATGAGGGGCGAGAGGACCCGTAATAGAAATCTCG
CCCGGCTGGAAACCTGCGTTCCGCGTGCCCAACAAACGCCACATCTCCTCAGTGGCTAAACGAGGGAAAA
ACAATGTTTCGTGGCGGCGGTACACTTTACACCGTGGCTGGGCGCCAAACACCTGTAATTGTGCAGGGGC
CCAAATTTCGTGCTAACTTTCGTTGCGGGAGAGGGTGGAACCTCTTCTTCTTTTCTACTACGTGTCCAGA
GGGGCACCGAGGCTGGGGTCCCATCCGCGAGGATGGGGGGCACCCACGAGTCTTTCCAAACTGCT

>OK018178.1 Teosinte-associated betaflexivirus isolate P1D8S49, complete genome
GCAGCACAATTCCCTGTAGCTAGTACCAAGTGTAAACTACCATAACACAGATGGAGTTTTACAGGACAGC
TGCAGAGAATTTCCTTGACATTCTAACCCCTGAGCAAAGAGATGAACTGAGAATCCCACTGGTAGAGGAA
TTTAAACTTGCCTCCAGAAGGGATTGGATTCATTACTCTTATGCTCTGAAGAATAGTCAAAGGAGGTTGC
TCAGTCAACATGGTATCCCTCTACATCCAAACGGATGCTTACCACATCCACATCCGGCTTCAAAGACCAT
CGAAAATTTTCTTTTACATGGGGTGCTTTCACCTCTTGTAAAGGAAAAGGTTACATTGAATAATGAGAGA
ATCACGCTTTTCAGTATGAAACAACGAAAATTTGATGCATTTAGAGCCTCCAATGCCATATTTGACGATT
TGAATTGTGACCTTTTCAATCGCCTATACCATACAAACGACGTTACCAGATATGGGAGTGCTTGGCCTAC
CCAGTGCAAGGGTGAAAATTATAATAGATTCTTTAACGTGCATCGACGGAAGTTTGAAGATAGGGTGGTT
GTCATTCATGATGAGATACATCATTGGGGGGTTTCTGACCTGGTGAGCTTTTTGGATGTTGCTAAACCAA
AAACAGTTTTTGCCACAGCAGTGTACCCCGTTGAAGTCATGTTTGGAAGCCATACGTCATTGAATCCATG
CCTTTATGAGTTTGACATTAAAGGTGATACATTGCTCTATTACCCAGATGGAAAAACTGATGCCAAGTAT
GAACAGAAAAGAACTTTTGACATCTTGAGAATGGGTGTGGTTCATGCTAATAGAACCTACTGCATATCGG
TTGCAAGGTCAATAGGAAGTCACCATCTAATTGTAATCGAAAGGGGCAATTTGGCTGTGGAGAGATTTAG
GGTGTTCAACGATTTCACACACTATAACCCATTAATTAATGGAGAGATGTTATCAAGAGTCAGGAATTTA
AGAATTGATAGAGTTAAACTGACAAAGCTGATAATTTACATGATGTCCCTCAAAAAGCCAGACGTGGAGA
GTTCAATGGCTAAGTTAAGGCAGCTGTCAGATGACATTGATCCCTGTGAAATTATGCTTGCCAAGGAGAT
AGGCTCCATACTAGTTAAAAAAAATCTTGAGTTTGGTCATCGCTTTTTTGAGGCTTTCCACGAGTGGCTG
AGCTCAGGGTTTATAAAGGAGTCATTACTTATTAATGCCGGGGTGATTAATGGTGCCGATCTTTTACTAA
AGGCTTTTAAAAAGGAGTTCCAGCATAAATTTATCTTTGACCTTGAAGAGAGGGCCATTATAGTGGATGA
GTATGTGAGAATGGATTGTAATAACATAGTCGAGGTATTTGATTCCGTATGGATGGGGTCAAATATGTCT
GACATTATGGATCGACCCTCTATGAGGTATGAGCTCTTTGGATTCGAGCTTGATCACTCTGTGCTGGATC
TTAAGTTTTATGTGCTTCTTAATTCAGAGAGCCCTGATGACACAGTTAGGGAAATATTGATTCGGAAAGG
ACAGAGGCGAGCAAGGCGGCATGTGATAGTCATCTCCCGATCAAAGAAGTGGAGGTTAGGATGGAAGGAG
GCTGAAGAGAGAGGAAGGAGTGCCTCTGAAGCATCCTTCGATTCAGTAGAGTTTGAAAGAGAAATTTTAG
AGCTTGAAAAAAGGGAGGAGTTTAATATTTCAACCGTATTTAAAAGTGAGGAGGTGGAGGTTATGGAAAA
AATATCTAAAGAAGGTTGGAGTGGGAAGAACGTTTGTTTGATATCCGCCGTGGCTGACTCTGTTAAGATG
GATCCGATCAATTTGCTAACCTTTCTTTTTGCCTCAGATCCAGTTTATTGGGGATCTTGGCTCTTAAGAT
CAGAGGGGTCTGATGAACTAAGTGTATCGAAACTGGCTGATTACCTTAAAAGAGCGATTGTGATAGTTGA
AGGTGAGATAGAGCAGCTCTTTGGTGTTGAGAGTGAGGAGGAACCGATAATTCTTGATAAGAAAGGTGAT
CATGTTGTTTTGCGACTAAGGCAGGAGTGTATGTTTAATTTTAGCAGACCTAGCTTGGATGTCCGCCTTG
CTTTTGACAATGAGATAACAAGGTTACCGTACCATTCAAAGTTTATCTATGAAGTTAGCTATGAGAGGAC
CAAAATTTTGTCCAATGAATTCCACTGTGGTAGGGAAGGGGTCCTTTTTGGGGAAATCAAGGACAAAAAA
ATGCTTGACATCAAGAAGCACAGTGATTTTGATCAAGAGATTCATGTTGTCTCCGGTCTGCCAGGGTCTG
GTAAGAGTAAATTCATTGAAGGCTTTGTTAAGAGACTCAAGAGCAAGAAGATAATAGTGATATGTCCTCG
AAAAAAAATAAAGGAAATATGGGAGAAAAATGTGGACAATGTTGTTACTTATGAGGTCGCATTAACAAAG
AAAATGGACTCATATGATATCTTTATACTTGATGAATACACCCTGTATCCGCCAGGATATTTGGACATTG
TGATAGGCTCGAATGAAGGGAGGGATGTTAGATACTTATTAATTGGCGATCCCTTACAGGCAGCCTTTCA
TTCCTTTGAAAGCAGAAAACTAAATGAGATCAGCTTTAATGTTCTTAAAAACTTACAAGAGCTCAATTAT
TTATTTCTTTCTCATAGAATGGGAGGTTGGGCCAATTCTATATGGCCATTTGAGTGTGTCGGGCATTCAG
ATGAATGGAGATTCAACCATGTTGTCTTCCAGTCAATAGCCTCCTTATTTGGGGTGATTAAAGATGAGGC
AGAATTCCCAGAGGCATTTATCTGTGCCTCTTTTGATGATAAGGTGCTTGTACCTAAGAATTGCGGGGTT
CCAGTTATGACTTTTGGAGAATCGCAAGGAACAACTTTTGGGAAGGTTGGAATTATAGTTACAGGTAATT
TTAAGAAGGTTTCCAATAATCACTGGTTTGTGGCTCTTACAAGATCAAGAAGAGGGAACTGCTTCATTAA
TTACACTGACGTTGCGTTTGATGTTGTATTGAAACGAAACCCTGACACCCTCATATCTAGGTATTATAAC
AAAAGGATTTCAGAAGATTTTTTGAATGGTTTTGTAATCGAAAGTGGAGTTAGGAAGATGAACAGAGTGG
GGAATGTGAACATGAGAAGGTCTGACGTTGAAATCAAGTTGTCAGGGGATCCTTGGTTGAAACCATACAT
GTTTATAGGAAGATGTCCTGAAGCTCAAATTGTAAGAATGCAGAAAGCTTTTTTACCTGATCCAGAATTT
AAGACGCATCTTCCCCTCTGTGAAGAGAAGATTGACTTTGCTTCAATGTTTGAGGCCCTTAAAGCCAAGG
AGTTCCGAGAATTTAGGAGACCATATGATATGTCATCACAATTTATTGACCAGCAATCAAGGGAGCTGGA
ATGTGGGGCTGGGCAGCCTTTTTTATTTGAGTCAATCTATCCAAGGCACAGGAACTCCGATGAGGTAACT
TTCTGGGCTGCCGTGAGGAAGAGGTTAAGATTTTCAACTCCGGAGATAGAAAGAGAGAAGTATGAGAAGA
ACTTCAAATTTGGTAGATTGATCTTTGATAACTTCATAAAGTATGTGCCGATGGATAGTAACTTTGATGA
AAGGTTAATGTTAAAAGTGCAGGATGACTTTGAAATAACCAAGCTCAAAAAGAATGCTGGAACAATTGAG
TCACATGCCATGAGATCAAATAGAGATTGGGATGAACTAAACGTCTTTATATTCATTAAGACTCAATTAT
GCACTAAATTGGAAAAGAGGTTCTGTGACGCCAAGGCCGGGCAAACTCTTGCTTGCTTTTCTCATCTAGT
TCTCTGCCACTTTTCAAAGTGGTGTAGGTATATAGATGCAAAGCTGGACAAATGTCTATGTGAGTTGAGA
AGAGCAGGAAAGCATGATTTTTATGTACATACGAGGAAAAACTTCGATGAACTTAACGAATGGGTTAAAA
GACAAAGTTTTAATGGGGTTTGCACTGAATCTGACTATGAGGCCTTTGATGCTTCACAAGACTCAATAAT
TTTGGCATTTGAGGTCTGCATAATGGTTCACTTTGGTTTTCCGGTTGAAATAGTGGAAGCTTACAAGCAT
ATCAAATTTAATTTAAAATCCAAATTGGGGAGATTTGCCGTCATGCGGTTTACAGGGGAGTTTTGTACAT
TCTTGTTCAACACATTGGCTAATATGGCATTTACCTTCCTTAGGTATGACATGGATAAGGTCAAGTCAAT
ATGCTTTGCGGGTGATGATATGTGTGCTAATGGCAACTTAAGGGAGATCAGTGGGTTCGAGGAGGTCCTT
GGCAAGATGTCTCTCAAAGCTAAGGTATGTAGGACCAAATGCCCAACCTTCTGTGGGTGGAAGTTGACGA
AATATGGCATTGTTAAAGATAGCAGGTTGATCTGGGAGAGATTCATGGTGGCAAAGGAGAGGAATAACAT
GAGAGAGTGCCTCGAATCCTACACATTAGAGGCTTGTTACGCCTATGAATTAGGTGATAGGCTCTTTAAT
GTCCTTGATGAAGAGCAGCTGAGGTATTATTACTTAATCAACAGATTAATTGTCAAGAATCTGAGCAGCT
TGCCAGTCTCAATAGCCTCGGTTTATTCAAAGATCAATTACCTGCTTGATGGAAGTGATAAAGGTGAACA
AGTTTACGAAGGAGTTTGTGAGCTCTGGGAGCCTGACAGATCAGATTCCCAGTGGCTCAATTTACCGTGA
CGTGCCATGGATCGGAACAGGGGCAAGGGCAAAAATTGCCAAGCGAGAGATAAATGTGTCCTTGACCCCT
AAAGAAGGGGGGTACTCAATTGAAAATTTTCCCCTGCTAGACTCAGAGGACCTGATGTACTACAAGTCCG
CAGCTAAAAGTCACCCTTATGTTGATTTGGGTTGTGTCGTAATATCTATTAAAGGACTATTTAGAAGGAA
TGCTGGAGTTAAGGGAAGGCTGTTGCTTCTTGATGACATGTTTGATAACATTGAGCAGGCGAAGATATCT
GCCTTCAAATTCAATCTTGATGAAGGGTTTGCAGCCTTTGCTGTTTTTCCTGGGTATTCAATTGCAACGG
ACGACATGCAGTTACAGAGGTTGAGGCTAATAGTAGAATTTGAGAACCTAAATGTGAGATCAGGCTCATA
CAAGCCTGTAGCGATATCTGTTGGGCTCATTACAAAACTATCGACCACGGCATTTCCACCAAAGTCAAAA
TTGCTTGAAGAGAATGAGTTTGTTCACCAATCAATAGTTGGGTCACAGGTCTTGTCTTTGGAGGAGATCG
AGGGGAAGGATAAGCTTGCAGAGGAATTTAGGGTGCACAGTTTTGTTGATGGGTTGAAGATGCCAGAGGT
TGGCAGCAGCGTTAGAAGAAGGAAGGCACTATTGAGAACGCTGCCTCCGACACGTATTAAAACCTACACT
GCCGACGGTCCAACGGAAATGGGTGGAATCGTTTCAGAGGTGTCAGAAGGTGCGTCAATAATGAGTGGTG
ATCGGGAGGAAGTAGGGTCCTCCTCTATGAGGGTGATCTCGACTGCTATGGAGGCTGGTACTCAGCTGGA
GGTCAGAAGAAAATCAGTTTCATCTGTTCTACTGTAAACTAGTTAGGTCCGGCTGAAGACGTTAAACTTA
GCGCTTGTGGGGAGGCAACCCCATTTAGAGGAGTAGTTAGGTTTTCCCGGTGTTGATGATTCAAGATGTC
GATAGAGAGAAGGATTCAAGAGAACAGAAGGACGTTGGTGTCTGAAATTGTTGTTCAATTTGTGATGGGG
GGAAGGGCTGTCCCAGAAGGGGATAGGATAGATGTGTGCAATGGTATAATCAAAGATGTATTTGGAAACA
TAGCCTACTATCAGGCTTCTGAATCAACAATTTTCCCTGATGTGAGAAAACAAGTGACCATTATCTGTGA
TAGGGAAGAAGGGGATGATGTGACCCACAACTTTGAATACAATTTAAACAGGCTAACTAATGTGCTCAGG
TCTGCCCCAGTTGTTAGCCAAGAACCTTTAATTAAAGGGGCGACTTTCAGACAGCTTTGCATTCCGTTTG
CGGAAATGGCAAAGGTGTCTTTGAGGGAGCAGTACACATTGGATGGCACTGTATCTGCACTTGCATTAAC
CCTACCAAAAATATGTGAAAGGGCACCATGGGTGGCCTTTGATTTCAACAGGGGGTTAAATTATAGGCTT
TTGACAAGTGCTGAAAGGTCAGTAATTCAGAACCTGAATAGAAGGCTTCTATCCACAACATTGAAGGTCA
ACATTGGGGAAGCCCAGGCTGACGGTGCGACTAGTGATGTGGCCGCATAGTCAACAATTTAATAAACATC
ACCTCTGGGGTTTCTATACCCTCTTTACCCAGAGTATTGTAGAGGGCTAGCTTTATCTAAGGGGCTAAGT
TTAAGTTTTAAAAATTTTCTTAAAAAA

>MZ852895.1 North American maize-associated mastrevirus, complete genome
TAATGTTACGCCCATTGCTTTTTGGCGCCCTTTAGTGGGTGCGATGGTCTGATGTTAAAGATAGGTTGCT
TCGTAACGAAGTTATTTAACAATGGAAAGCGGACATCTCCCCCAGATTTCGCCGCCGGTATATTTCACGG
GTTCTGCTTCTCAAGGTACGAACCCTACCGGAGTCGGAAACGACGCGGCTTGGAGGTTCCTTGTCCTGTT
CTTAGCTTGCGCGGCCGTGTCGCTTGGGATCATCATATTTCTCTATAAGACGTGTTTGAAGGACCTTCTT
CTCACCTGGAGGGCTCGCCGGAGCAGGACAGTAACCGAGTTAGGGTTCGGTGCCACACCTCAGAGACCCG
CTGGAGCAGCACCTCCTCAAGTGGGCCAAGTCGGACCTTACGGGTAGCGGTCGCGTAACTCATTTTTCGA
GTTACGCTGCTTTTCGCAGGGTGCTTGAGGCTTCTAGGTCCTACCCAGGGACCGTACCGAAGCCCAGCGG
CCCAGTTATGTCGAGGCCTTTGAAGAGAAAGAGGGAGACAAAGTACCGCTGGCCCGAAGCGGCAGCGAAG
AAGGGGTTTACCCCAGCGAACTCGAAGTGGGTTCGCGGTTATAAGCCCCCACAAAGGAGGCCTTCTTTGC
AGGTTCAGACGTACAGTTTGTATGGGAACTCCACCTGGAACATTACCAAGGGGGGTCAGGTTGATTTACT
GACGTCATATTCGCGTGGGTCTGACGAGGCCCAGCGCCATTCATCAGAGACTATGACGTATAAGTGTGGC
CTTGATTTATTTTTTTATTTAAAACCAGAAAGGTTGAATTCAGTGTGGCGTGCATGGAATGTGGCATGGT
TGATTTACGATGCTGCACCGATTGGAGCTATGCCCACGACCAAAACCATTTTTGGTTATCCGGACGAGCT
AACTGACCATCCATATACTTGGAAGGTGGCAAGAGAAGGTGTACATCGTTTTGTCATTAAACGCAGGTGG
GTGTTCAAGCTCGAGTCAAACGGGATTCCAAACGGGACAACGTTTACTACGAGTGGCGGAGGGACTCCTT
GTCAGAAGAGCCTGTACTTCTCGAGGTTCGTAAAGAGGTTGGGTTGCAGAACCGAGTGGAAGAACAGTGT
AAACGGCCAGATCGGAGATATAAAGAACGGAGCATTGTATATAGCTGTAGCACCAGGTCTGGGTAACGCA
TTCGATGTGGTTGGCACTTGCCGCATGTACTTTAAATCTACTGGTAATCAGTAGATATGTAAACATGTAA
TAGTTTATAAATACACGAAGTTTATTTATCATCCAGCTAACAAGGCTGGGCTTCACACAAATTACAATAC
AAATACCACAAAGGAGGACAGGACAAAGCAGGCGGCTAAGGGCGCGCATGGGGCAACACAAAACCCCGGA
CAAACTTTAGTAACTTGCAGCTACTGGCTTCAAGCTTCGGAGTAGAAGCGTTCTCCTGGATACATGATGT
AGATGTCGCAATTCGCTTCTAGATACTCCTTCTGCTCGGGCTTCATTGCCTCCAGCCAGCACTCGTCAGA
ATTGACGAGTATTATAGATGGGATGCCTCCTTTGATTACTTTCTTCTTGCCATATTTAGGGTTCACGGTG
TAGTCCCGCTGTGCCCCTACAAGCTGCTTCCAACACGGACAGTATTTAAATGGAATATCATCAATTACAT
TATATTTAGCTGCTTCGTCGTAGCAGCTAAAGTCGACGTTGTTTTGCCAGTAGTTGTGAGGTCCTAGGCT
CCGGGCCCAGGTAGATTTGCCAGTCCGTGTTGGCCCGCAGATGTAGAGGCTCCGGGGTCGTAGCCCCACC
TGAGTTGGTTCCTGGTGAAGTCGGCCATCCAGTTTAGGTCGACCTTTGCCTGATCTATGGTGGTGCAGCT
TGAAGAGTGACCCAGGAAGTAGGCAGTTTCACTTACAGTGTACAGGCGTTCAGCGAAGCCATTGATGGTT
TCAGTGCAATTGAGGTTTAACTCGCTCTGTGGGAAAGGGTTCTGGTAGGGCGCTGGAGTGTCTGGGAAGA
GACGTTTGGCTGAGTACTCGAATGCTTGAAGTTTGGTGGCCCAGTCATAGGGCATGTTATCACGGACCAT
TCCGAGATAATCTTCTCTGGAGGAGGCGTTGTTGATGATGTCTCGCATTGTGTTGTTGTTCGTGAGGATT
GTGGATTTGGTGGCACCTCTGCTGGTACGTACGAGATTACCTCTGGTGAACTGGGAAATGATGTTTTTGG
TGATGTATGTTTGGACGAGTTGAGCGGATCGGCAAGTTTGAATATTAGGATGGTGGTCACAGATATCGAA
ATACCGTGAATTATGAGTCGATATTTCGTGTTCCACTTGGAATAGGACGTGCAGGTGATAAGAACCATCC
TCGTGAGATTCACGAGTAACCATGATATACAGAGGGCCATACCGTGCGTTTGCAGTCCAGAGGTATTCGC
CGACGACATTTGGCTCAAGTGGGCAGCGTGGATAGGTTAGGAAAATATTCTTGGATTTGAAACGGAAACG
TCCCGAGGGGGAACGGCCTCTCGGAGTTGGCGGGGTTGGAGTCGTTACGGGCGAGGGGTTGTTAACCCAT
ACAGGTGGACTGTCAGCTTGGTATACACTCTCTCCTCCGTTGTCCATGAGGTTTGCAAACTCTATCGTAT
AGTAACAGCTTCTGCCTCGCCCATCCGCTTTATAGCGGTTCATCTGGGCCGTCCGGCCCAGCCCGATAGC
AAAAAGCAATGGGCGAGTGCGGCAGCACAA
