# Supplementary figures and images for: Metagenomic identification of novel viruses of maize and teosinte in North America
Source: BMC Genomics. 2022 Nov 23;23:767. doi: 10.1186/s12864-022-09001-w (PMC9685911; doi:10.1186/s12864-022-09001-w)

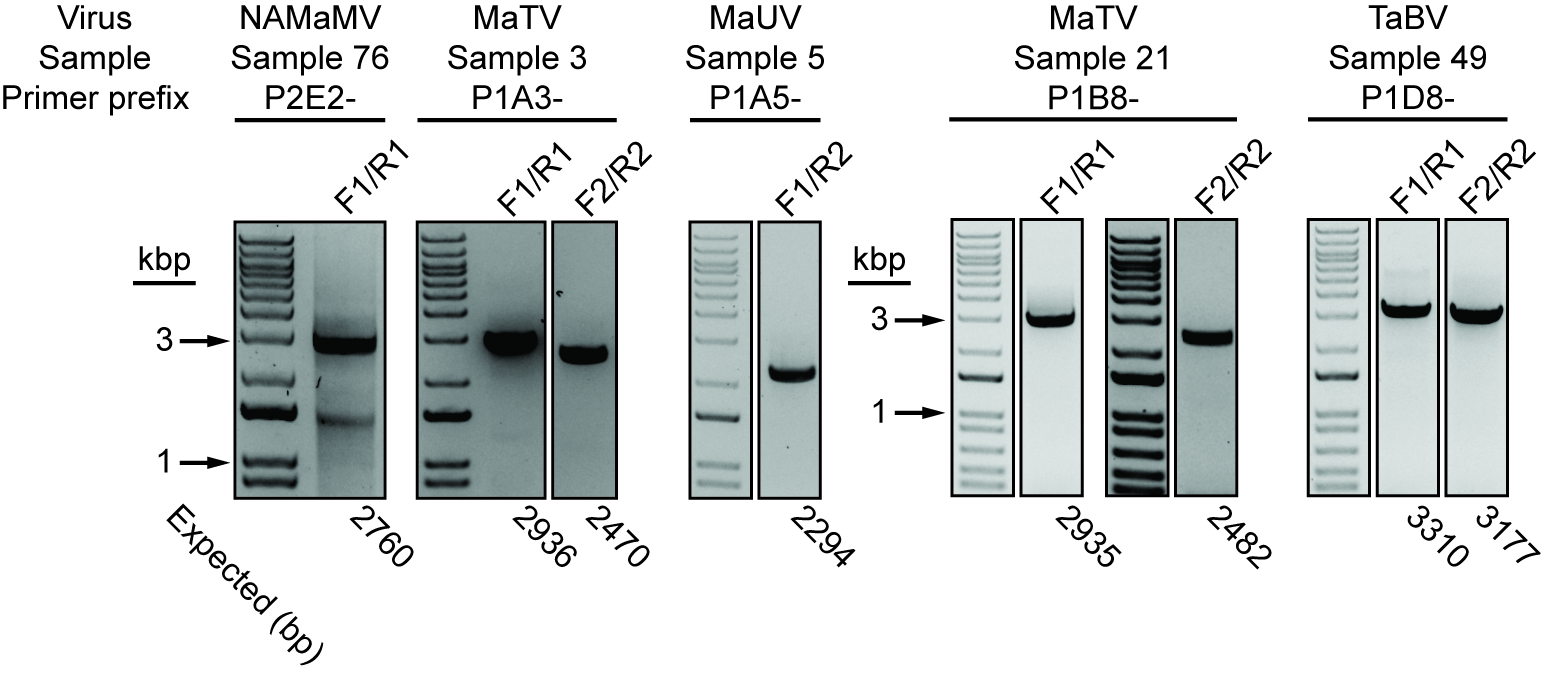

Supplement: Supplementary file 5 — Additional file 5: Supplementary Fig. 1. Amplification of novel viruses identified by RNAseq for validation of presence and sequence. Nucleic acids were isolated from leaf samples identified by RNAseq to contain novel viruses. Resulting DNA or cDNA was used for amplification of viral sequences by primers designed from assembled contigs (Supplementary Table S2). Subsequent to amplification, the fragments of interest were gel extracted and cloned into pUC19 for Sanger sequencing. [file 12864_2022_9001_MOESM5_ESM.tif]
