# Supplementary material for: Metagenomic identification of novel viruses of maize and teosinte in North America
Source: BMC Genomics. 2022 Nov 23;23:767. doi: 10.1186/s12864-022-09001-w (PMC9685911; doi:10.1186/s12864-022-09001-w)
Supplement: Supplementary file 6 — Additional file 6: Supplementary Fig. 2. Annotation of putative nucleotide sequence elements of NAMaMV. The genome sequence of NAMaMV highlighted with corresponding labels and colors for open reading frame start and stop codons, as well as the presumed Rep-associated intron in orange text. [file 12864_2022_9001_MOESM6_ESM.docx]

**Supplementary Figure 2. Annotation of putative nucleotide sequence elements of NAMaMV**

Virion-strand origin of replication Movement protein start codon

TAATGTTACGCCCATTGCTTTTTGGCGCCCTTTAGTGGGTGCGATGGTCTGATGTTAAAGATAGGTTGCTTCGTAACGAAGTTATTTAACAATGGAAAGC [100]

GGACATCTCCCCCAGATTTCGCCGCCGGTATATTTCACGGGTTCTGCTTCTCAAGGTACGAACCCTACCGGAGTCGGAAACGACGCGGCTTGGAGGTTCC [200]

TTGTCCTGTTCTTAGCTTGCGCGGCCGTGTCGCTTGGGATCATCATATTTCTCTATAAGACGTGTTTGAAGGACCTTCTTCTCACCTGGAGGGCTCGCCG [300]

Movement protein stop codon

GAGCAGGACAGTAACCGAGTTAGGGTTCGGTGCCACACCTCAGAGACCCGCTGGAGCAGCACCTCCTCAAGTGGGCCAAGTCGGACCTTACGGGTAGCGG [400]

Capsid protein start codon

TCGCGTAACTCATTTTTCGAGTTACGCTGCTTTTCGCAGGGTGCTTGAGGCTTCTAGGTCCTACCCAGGGACCGTACCGAAGCCCAGCGGCCCAGTTATG [500]

TCGAGGCCTTTGAAGAGAAAGAGGGAGACAAAGTACCGCTGGCCCGAAGCGGCAGCGAAGAAGGGGTTTACCCCAGCGAACTCGAAGTGGGTTCGCGGTT [600]

ATAAGCCCCCACAAAGGAGGCCTTCTTTGCAGGTTCAGACGTACAGTTTGTATGGGAACTCCACCTGGAACATTACCAAGGGGGGTCAGGTTGATTTACT [700]

GACGTCATATTCGCGTGGGTCTGACGAGGCCCAGCGCCATTCATCAGAGACTATGACGTATAAGTGTGGCCTTGATTTATTTTTTTATTTAAAACCAGAA [800]

AGGTTGAATTCAGTGTGGCGTGCATGGAATGTGGCATGGTTGATTTACGATGCTGCACCGATTGGAGCTATGCCCACGACCAAAACCATTTTTGGTTATC [900]

CGGACGAGCTAACTGACCATCCATATACTTGGAAGGTGGCAAGAGAAGGTGTACATCGTTTTGTCATTAAACGCAGGTGGGTGTTCAAGCTCGAGTCAAA [1000]

CGGGATTCCAAACGGGACAACGTTTACTACGAGTGGCGGAGGGACTCCTTGTCAGAAGAGCCTGTACTTCTCGAGGTTCGTAAAGAGGTTGGGTTGCAGA [1100]

ACCGAGTGGAAGAACAGTGTAAACGGCCAGATCGGAGATATAAAGAACGGAGCATTGTATATAGCTGTAGCACCAGGTCTGGGTAACGCATTCGATGTGG [1200]

Capsid protein stop codon

TTGGCACTTGCCGCATGTACTTTAAATCTACTGGTAATCAGTAGATATGTAAACATGTAATAGTTTATAAATACACGAAGTTTATTTATCATCCAGCTAA [1300]

CAAGGCTGGGCTTCACACAAATTACAATACAAATACCACAAAGGAGGACAGGACAAAGCAGGCGGCTAAGGGCGCGCATGGGGCAACACAAAACCCCGGA [1400]

Rep-associated protein stop codon

CAAACTTTAGTAACTTGCAGCTACTGGCTTCAAGCTTCGGAGTAGAAGCGTTCTCCTGGATACATGATGTAGATGTCGCAATTCGCTTCTAGATACTCCT [1500]

TCTGCTCGGGCTTCATTGCCTCCAGCCAGCACTCGTCAGAATTGACGAGTATTATAGATGGGATGCCTCCTTTGATTACTTTCTTCTTGCCATATTTAGG [1600]

GTTCACGGTGTAGTCCCGCTGTGCCCCTACAAGCTGCTTCCAACACGGACAGTATTTAAATGGAATATCATCAATTACATTATATTTAGCTGCTTCGTCG [1700]

RepA protein stop codon

TAGCAGCTAAAGTCGACGTTGTTTTGCCAGTAGTTGTGAGGTCCTAGGCTCCGGGCCCAGGTAGATTTGCCAGTCCGTGTTGGCCCGCAGATGTAGAGGC [1800]

Rep-associated intron

TCCGGGGTCGTAGCCCCACCTGAGTTGGTTCCTGGTGAAGTCGGCCATCCAGTTTAGGTCGACCTTTGCCTGATCTATGGTGGTGCAGCTTGAAGAGTGA [1900]

CCCAGGAAGTAGGCAGTTTCACTTACAGTGTACAGGCGTTCAGCGAAGCCATTGATGGTTTCAGTGCAATTGAGGTTTAACTCGCTCTGTGGGAAAGGGT [2000]

TCTGGTAGGGCGCTGGAGTGTCTGGGAAGAGACGTTTGGCTGAGTACTCGAATGCTTGAAGTTTGGTGGCCCAGTCATAGGGCATGTTATCACGGACCAT [2100]

TCCGAGATAATCTTCTCTGGAGGAGGCGTTGTTGATGATGTCTCGCATTGTGTTGTTGTTCGTGAGGATTGTGGATTTGGTGGCACCTCTGCTGGTACGT [2200]

ACGAGATTACCTCTGGTGAACTGGGAAATGATGTTTTTGGTGATGTATGTTTGGACGAGTTGAGCGGATCGGCAAGTTTGAATATTAGGATGGTGGTCAC [2300]

AGATATCGAAATACCGTGAATTATGAGTCGATATTTCGTGTTCCACTTGGAATAGGACGTGCAGGTGATAAGAACCATCCTCGTGAGATTCACGAGTAAC [2400]

CATGATATACAGAGGGCCATACCGTGCGTTTGCAGTCCAGAGGTATTCGCCGACGACATTTGGCTCAAGTGGGCAGCGTGGATAGGTTAGGAAAATATTC [2500]

TTGGATTTGAAACGGAAACGTCCCGAGGGGGAACGGCCTCTCGGAGTTGGCGGGGTTGGAGTCGTTACGGGCGAGGGGTTGTTAACCCATACAGGTGGAC [2600]

RepA and Rep-associated protein start codon

TGTCAGCTTGGTATACACTCTCTCCTCCGTTGTCCATGAGGTTTGCAAACTCTATCGTATAGTAACAGCTTCTGCCTCGCCCATCCGCTTTATAGCGGTT [2700]

CATCTGGGCCGTCCGGCCCAGCCCGATAGCAAAAAGCAATGGGCGAGTGCGGCAGCACAA [2760]
