# Supplementary material for: Metagenomic identification of novel viruses of maize and teosinte in North America
Source: BMC Genomics. 2022 Nov 23;23:767. doi: 10.1186/s12864-022-09001-w (PMC9685911; doi:10.1186/s12864-022-09001-w)
Supplement: Supplementary file 7 — Additional file 7: Supplementary Fig. 3. Annotation of amino acid motifs predicted in putative NAMaMV open reading frames. Putative open reading frame translations are highlighted with corresponding labels and colors with pfam identification numbers identified by Blastx analysis. [file 12864_2022_9001_MOESM7_ESM.docx]

**Supplementary Figure 3. Annotation of amino acid motifs predicted in putative NAMaMV open reading frames**

**Movement protein**

Pfam01708 [1]

MESGHLPQISPPVYFTGSASQGTNPTGVGNDAAWRFLVLFLACAAVSLGIIIFLYKTCLKDLLLTWRARRSRTVTELGFGATPQRPAGAAPPQVGQVGPYG* [101]

**Capsid protein**

Pfam00844 [1]

MSRPLKRKRETKYRWPEAAAKKGFTPANSKWVRGYKPPQRRPSLQVQTYSLYGNSTWNITKGGQVDLLTSYSRGSDEAQRHSSETMTYKCGLDLFFYLKP [100]

Pfam06370 (DUF1069) [1]

ERLNSVWRAWNVAWLIYDAAPIGAMPTTKTIFGYPDELTDHPYTWKVAREGVHRFVIKRRWVFKLESNGIPNGTTFTTSGGGTPCQKSLYFSRFVKRLGC [200]

RTEWKNSVNGQIGDIKNGALYIAVAPGLGNAFDVVGTCRMYFKSTGNQ* [248]

**RepA protein**

Rep catalytic domain pfam00799 [1] RCR motif I [2] RCR motif II [2]

MDNGGESVYQADSPPVWVNNPSPVTTPTPPTPRGRSPSGRFRFKSKNIFLTYPRCPLEPNVVGEYLWTANARYGPLYIMVTRESHEDGSYHLHVLFQVEH [100]

Geminivirus Rep Sequence [3] RCR motif III [2]

EISTHNSRYFDICDHHPNIQTCRSAQLVQTYITKNIISQFTRGNLVRTSRGATKSTILTNNNTMRDIINNASSREDYLGMVRDNMPYDWATKLQAFEYSA [200]

LXCXE retinoblastoma binding domain [4]

KRLFPDTPAPYQNPFPQSELNLNCTETINGFAERLYTVSETAYFLGHSSSCTTIDQAKVDLNWMADFTRNQLRWGYDPGASTSAGQHGLANLPGPGA* [297]

**Rep-associated protein**

MDNGGESVYQADSPPVWVNNPSPVTTPTPPTPRGRSPSGRFRFKSKNIFLTYPRCPLEPNVVGEYLWTANARYGPLYIMVTRESHEDGSYHLHVLFQVEH [100]

Rep protein central domain pfam08283 [1]

EISTHNSRYFDICDHHPNIQTCRSAQLVQTYITKNIISQFTRGNLVRTSRGATKSTILTNNNTMRDIINNASSREDYLGMVRDNMPYDWATKLQAFEYSA [200]

dNTP binding domains

KRLFPDTPAPYQNPFPQSELNLNCTETINGFAERLYTVSETALRPRSLYICGPTRTGKSTWARSLGPHNYWQNNVDFSCYDEAAKYNVIDDIPFKYCPCW [300]

KQLVGAQRDYTVNPKYGKKKVIKGGIPSIILVNSDECWLEAMKPEQKEYLEANCDIYIMYPGERFYSEA* [369]

**References**

1. El-Gebali, S., et al., *The Pfam protein families database in 2019.* Nucleic Acids Res, 2019. **47**(D1): p. D427-D432.

2. Koonin, E.V. and T.V. Ilyina, *Geminivirus replication proteins are related to prokaryotic plasmid rolling circle DNA replication initiator proteins.* J Gen Virol, 1992. **73 ( Pt 10)**: p. 2763-6.

3. Nash, T.E., et al., *Functional analysis of a novel motif conserved across geminivirus Rep proteins.* J Virol, 2011. **85**(3): p. 1182-92.

4. Arguello-Astorga, G., et al., *A novel motif in geminivirus replication proteins interacts with the plant retinoblastoma-related protein.* J Virol, 2004. **78**(9): p. 4817-26.

5. Gorbalenya, A.E. and E.V. Koonin, *Viral proteins containing the purine NTP-binding sequence pattern.* Nucleic Acids Res, 1989. **17**(21): p. 8413-40.
